# Comparison of multiple arterial grafts vs. single arterial graft in coronary artery bypass surgery: a systematic review and meta-analysis

**DOI:** 10.3389/fcvm.2025.1555242

**Published:** 2025-03-27

**Authors:** Qiuju Ding, Qingqing Zhu, Lichong Lu, Xiaofeng Cheng, Min Ge

**Affiliations:** ^1^ Department of Cardio-thoracic Surgery, Nanjing Drum Tower Hospital, The Affiliated Hospital of Nanjing University Medical School, Nanjing, China; ^2^ Department of Cardio-Thoracic Surgery, Nanjing Drum Tower Hospital, Clinical College of Nanjing University of Chinese Medicine, Nanjing, China

**Keywords:** multiple arterial graft (MAG), single arterial graft (SAG), coronary artery bypass graft (CABG), randomized controlled trials (RCTs), survival

## Abstract

Observational studies and randomised controlled trials (RCTs) have yielded conflicting results regarding the outcomes of multiple arterial grafts (MAG) vs. single arterial grafts (SAG) in coronary artery bypass graft (CABG) surgery. We conducted a comprehensive search across multiple databases for RCTs that directly compared MAG and SAG. The clinical outcomes assessed included all-cause mortality, cardiac-specific mortality, myocardial infarction (MI), repeat revascularization, stroke, sternal wound complications, and major bleeding. Outcomes were measured using hazard ratios (HR), relative risks (RR), and the corresponding 95% confidence intervals (CI). Eighteen RCTs involving 10,143 patients were included in the analysis. The follow-up period ranged from 6 months to 12.6 years, and the average age of the patients across the studies ranged between 56.3 and 77.3 years. MAG and SAG did not differ significantly in terms of the incidence of sternal wound complications, major bleeding, or stroke following CABG. However, the MAG group demonstrated a lower risk of all-cause mortality, cardiac mortality, MI, and repeat revascularization compared with the SAG group. MAG was associated with higher survival, lower risk of MI, and fewer repeat revascularization. Nonetheless, there were no significant differences in the incidence of sternal wound infections, major bleeding, and stroke between MAG and SAG.

## Introduction

Coronary artery bypass grafting (CABG) is the surgical treatment of choice for severe multivessel coronary artery disease (CAD). Single arterial grafting (SAG) is the standard of care for patients undergoing CABG. Nonetheless, multiple arterial graft (MAG) is gaining popularity (1, 2), and there is debate regarding what patients benefit from MAG.

Revascularization with a left internal thoracic artery (LITA) graft to the left anterior descending artery is well established (1, 2). The radial artery (RA), right internal thoracic artery (RITA), and saphenous vein (SV) are grafts routinely used as the second conduit. Although a *post hoc* analysis of the SYNTAXES trial demonstrated the superiority of MAG over SAG for patients undergoing CABG (3), several surgeons favour the use of the SV because RCTs have failed to demonstrate a survival benefit of MAG over SAG, despite differences in vessel patency (4, 5). Possible reasons for this finding include underpowered studies (6) or inconclusive results due to discrepancies between the treatment allocated and the treatment received in the Arterial Revascularization Trial (ART) (5). To overcome the limitations of the ART, the ROMA trial (7), which is underway, will compare all MAG approaches and SAG without imposing on the surgeon which graft configuration should be adopted.

Although previous meta-analyses demonstrated the survival benefit of MAG, most included studies analysed observational data. Further, other studies evaluated single MAG configurations (8), or failed to provide as-treated data (9), or did not perform sensitivity analyses (10), or did not evaluate clinical outcomes such as wound infections, stroke, and myocardial infarction (MI) (9).

This study conducted a meta-analysis of RCTs, including all MAG approaches—bilateral internal thoracic artery (BITA), left internal thoracic artery (LITA)+RA, and BITA/LITA+RA)—to comprehensively and systematically compare the clinical outcomes of MAG and SAG. The primary outcomes were long-term survival and cardiac mortality. The secondary endpoints were repeat revascularization and MI. Postoperative complications included sternal wound infections (SWIs), stroke, and bleeding.

## Results

### Study selection and baseline characteristics

**Figure 1:**
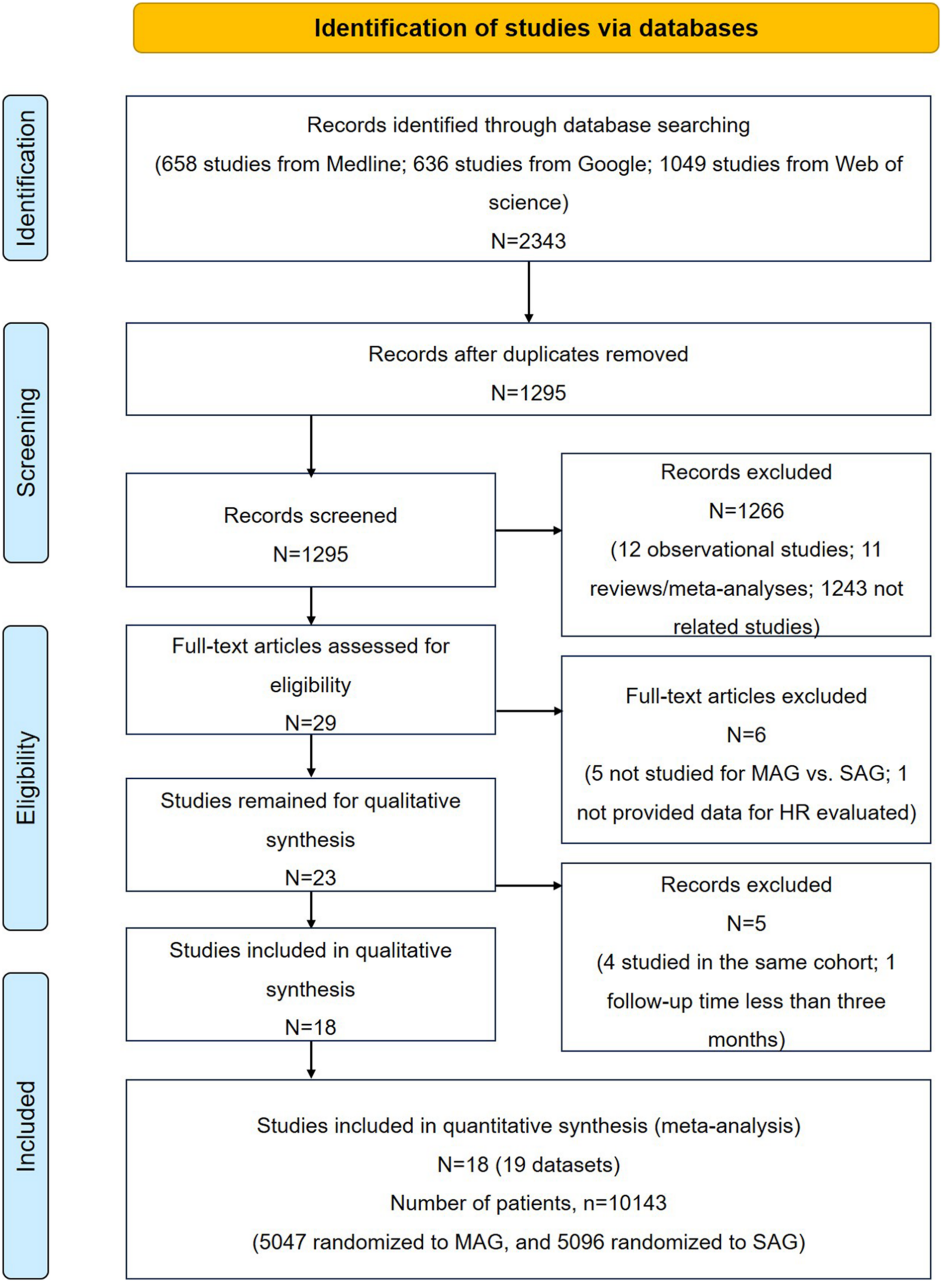
Flow chart for study selection.

The study selection process is illustrated in Figure. 1. A total of 2,343 articles were initially included after searching PubMed, Google, and Web of Science databases. After removing 1,048 repetitive articles, we further excluded 1,266 records based on the title and abstract, including 12 observational reports, 11 reviews/meta-analyses, and 1,237 unrelated articles. Among the remaining 29 records, five articles did not compare MAG and SAG; one articles did not provide the estimated HR; four studies researched the same cohort; one study had a follow-up period of less than 3 months. Thus, 18 studies were included in this meta-analysis. Of which, sixteen was RCT studies (4, 6, 11–24), and two studies were *post hoc* analyses of observational data from randomised trials that evaluated alternative treatments, including the subsets from SYNTAX trail cited by Thuijs (3) and cited by EXCEL trials Thuijs (25). Given that both subsets were relevant to our research aim, and two previously comprehensive meta-analyses of RCTs, conducted by Changal et al. (26) and Magouliotis et al. (9), also included these two subsets as RCT group. Thus, we decided to incorporate these subsets into our meta-analysis.

The included studies were published from 1990 to 2022 and were conducted in the UK (19), Italy (21), Australia (4, 22), USA (3, 6, 17, 20, 23–25), Canada (15), Russia (12), Korea (13, 16), Denmark (18), Serbia (14), and multinational (11). The total sample size was 10,143 patients (MAG: 5,047; SAG: 5,096). Among the 5,047 patients assigned to the MAG group, 1,440 underwent RITA and LITA grafting, 850 underwent RA and LITA grafting, and 2,757 underwent RA/RITA and LITA grafting. The LITA to the LAD approach was used in all patients. The average age ranged from 56.3 to 77.3 years. Among these studies, some of them provided data on gender and comorbidities (Table 1; Supplementary Table S5). Among these studies, the sex distribution and the proportion of preoperative complications such as hypertension, diabetes mellitus, previous MI and COPD was similar between the two groups. The MAG group has a lower prevalence of obesity and peripheral arterial disease, but a higher incidence of hyper lipidaemia (Supplementary Table S6). The number of grafted vessels was similar across the groups. A total of 2,111 patients (20.8%) underwent off-pump CABG, with a similar distribution between the MAG and SAG groups. The mean follow-up period ranged from 0.5 to 12.6 years. Nine studies (2,234 MAG, 2,081 SAG) had follow-up periods of less than 5 years, six studies (836 MAG, 818 SAG) had follow-up periods of 5–10 years, and three studies (1,978 MAG, 2,197 SAG) had follow-up periods longer than 10 years. There may be some biases owing to patient selection, interventions, and outcomes reporting in our study. The assessment of the risk of bias is shown in Supplementary Figure S1.

**Table 1:** Baseline characteristics of the studies included in the systematic review and meta-analysis.

Study	Country	Group	Number, *n*		Mean age, *n* (%) or (IQR) or mean ± SD		Male, *n* (%)		Urgent surgery, *n* (%)		Cardiopulmonary bypass on pump, *n* (%) or mean ± SD		Off-pump, *n* (%)		Number of grafts, mean ± SD		Follow up, years
			SAG	MAG	SAG	MAG	SAG	MAG	SAG	MAG	SAG	MAG	SAG	MAG	SAG	MAG	
Morris, 1990 (24)	USA	RITA MAG vs. SAG	420	643	60 ± 10	60 ± 10	NR	NR	195 (46.4)	304 (47.3)	Y	Y	–	–	NR	NR	4.0
Myers, 2000 (23)	USA	RITA MAG vs. SAG	81	81	62.80	62.60	61 (75)	63 (78)	NR	NR	133.50	135.90	–	–	NR	NR	5.0
Buxton, 2003 (22)	Australia	RA MAG vs. SAG	80	73	73.2 (64.0–82.4)	72.9 (62.3–83.5)	67 (84)	63 (86)	9 (11)	5 (7)	Y	Y	–	–	3.2 (1.9–4.5)	3.3 (1.4–5.2)	5.0
Muneretto, 2003 (21)	Italy	RITA/RA MAG vs. SAG	100	100	68.0 ± 8	67.1 ± 9	75 (75)	73 (73)	NR	NR	81 ± 7	56 ± 9	–	–	2.9 ± 0.6	2.8 ± 0.8	1.0
Muneretto, 2004 (20)	USA	RA MAG vs. SAG	80	80	76.8 ± 2	77.3 ± 3	43 (54)	45 (56)	NR	NR	70 ± 11	56 ± 14	–	–	2.52 ± 0.2	2.47 ± 0.5	1.0
Collins, 2008 (19)	UK	RA MAG vs. SAG	60	82	59 ± 7	58 ± 6	58 (97)	79 (95)	NR	NR	95 ± 28	96 ± 28	–	–	3.3 ± 0.7	3.3 ± 0.6	5.0
Nasso, 2008 (6)	USA	RITA/RA MAG vs. SAG	202	601	69.7 ± 3.5	NR	120 (59)	345 (56)	NR	NR	Y	Y	–	–	2.57 ± 0.9	NR	2.0
Damgaard, 2009 (18)	Denmark	RITA/RA MAG vs. SAG	170	161	59 ± 8	59 ± 8	150 (88)	142 (88)	NR	NR	Y	Y	–	–	3.2 ± 0.9	2.9 ± 0.9	1.0
Goldman, 2011 (17)	USA	RA MAG vs. SAG	366	367	62 ± 8	61 ± 8	362 (99)	365 (100)	38 (10)	42 (11)	319 (86.9)	325 (88.8)	48 (13)	41 (11)	≥3 grafts: 77%	≥3 grafts: 82%	1.0
Song, 2012 (16)	Korea	RA MAG vs. SAG	25	35	74.6 ± 3.8	72.7 ± 3.2	14 (56)	17 (48.6)	NR	NR	–	–	Y	Y	NR	NR	1.0
Le, 2015 (15)	Canada	RITA/RA MAG vs. SAG	30	30	5 (18)^a^	4 (13)^a^	29 (97)	29 (97)	0 (0)	1 (3)	56 (44–66)	86 (61–110)	–	–	NR	NR	0.5
Petrovic, 2015 (14)	Serbia	RA MAG vs. SAG	100	100	57.1 ± 6.5	56.3 ± 6.1	73 (73)	73 (73)	NR	NR	Y	Y	NR	NR	3.14 ± 0.66	3.08 ± 0.66	8.0
Kim, 2018 (13)	SouthKorea	RITA MAG vs. SAG	112	112	64 (IQR: 59, 70)	63 (IQR: 56, 70)	83 (74)	91 (81)	NR	NR	–	–	112 (100)	112 (100)	4 (IQR: 3, 4)	4 (IQR: 3, 4)	8.0
Thuijs, 2018 (25)	USA	RITA MAG vs. SAG	688	217	66.1 ± 9.5	64.5 ± 9.3	521 (75.73)	186 (85.71)	NR	NR	81.6 ± 44.6	87.2 ± 44.4	197 (28.6)	74 (34.1)	2.2 ± 0.6	2.3 ± 0.5	3.0
Buxton, 2020 (4)	Australia	RA MAG vs. SAG	112	113	73.1 (60.5–80.7)	72.6 (61.0–83.5)	91 (81)	91 (81)	21 (19)	26 (23)	Y	Y	–	–	3.3 ± 0.7	3.2 ± 0.9	10.0
Fomenko, 2021 (12)	Russian	RITA MAG vs. SAG	385	387	66.8 ± 6.2	67.4 ± 5.9	231 (60)	243 (62.8)	NR	NR	249 (64.7)	234 (60.5)	136 (35.3)	153 (39.5)	3.0 ± 1.0	3.1 ± 0.8	5.0
Taggart, 2022 (dataset 1) (11)	Multiple	RITA/RA MAG vs. SAG	1,084	1,010	64.20 ± 8.92	63.42 ± 8.86	939 (86.6)	892 (88.3)	NR	NR	–	–	413 (38.1)	403 (39.9)	NR	NR	10.0
Taggart, 2022 (dataset 2) (11)	Multiple	RITA/RA MAG vs. SAG	1,084	390	64.20 ± 8.92	62.03 ± 8.95	939 (86.6)	333 (85.4)	NR	NR	–	–	413 (38.1)	175 (44.9)	NR	NR	10.0
Thuijs, 2022 (3)	USA	RITA/RA MAG vs. SAG	1,001	465	66.5 ± 9.2	62.3 ± 9.7	777 (77.6)	401 (86.24)	NR	NR	–	–	146 (14.5)	101 (21.7)	2.8 ± 0.8	2.8 ± 0.7	12.6

IQR, interquartile range; MAG, multiple arterial grafts; NR, not reported; RA, radial artery; RITA, right internal thoracic artery; SAG, single arterial graft; SD, standard deviation; UK, United Kingdom; USA, United States of America; Y, Yes.

^a^ Age ≥70 (years).

### Primary endpoints

**Figure 2:**
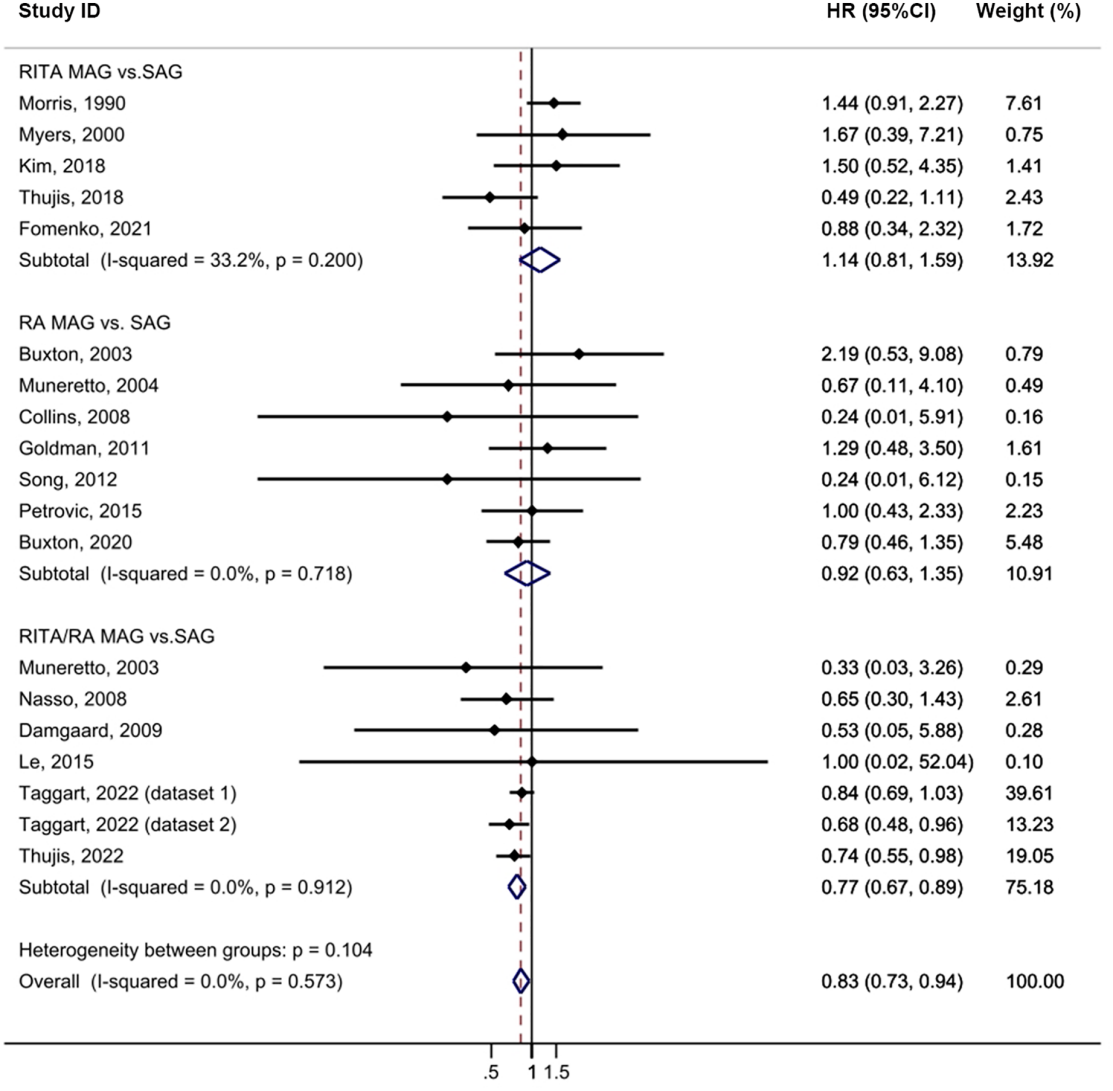
Forrest plot for all-cause mortality using intention-to-treat data. Horizontal lines represent 95% confidence intervals (CI). The rectangles represent the point estimate. The diamond represents the summary estimate (size of the diamond = 95% CI). The vertical line represents the reference of no increased risk.

In the intention-to-treat analysis, MAG demonstrated a significant improvement in long-term survival compared to SAG [HR = 0.83, 95% confidence interval (CI) = 0.73–0.94, *p* = 0.004; Figure 2] with low heterogeneity observed (I^2^ = 0%). The funnel plot did not reveal any publication bias among the studies (Supplementary Figure S2A). Prespecified subgroup analyses identified a notable difference only in the RITA/RA vs. SAG group (HR = 0.77, 95% CI = 0.67–0.89, *p* = 0.001). Furthermore, among the follow-up subgroups, studies with a longer follow-up period, > 10 years, exhibited a larger effect size (HR = 0.78, 95% CI = 0.68–0.90, *p* = 0.001), which may be partly attributed to the larger sample size. The meta-analysis results showed that MAG effectively reduced all-cause mortality (*p* = 0.004). In the *post hoc* subgroup, MAG significantly decreased all-cause mortality (*p* = 0.012). Nevertheless, in the group of pure RCTs, MAG showed a trend towards reducing all-cause mortality, but the results did not reach the statistical significance (*p* = 0.053; Supplementary Table S12). Sensitivity analysis revealed that, after excluding the study by Morris (24), the result became more statistically significant (HR = 0.79, 95% CI = 0.70–0.91, *p* = 0.001; Supplementary Table S10).

**Figure 3:**
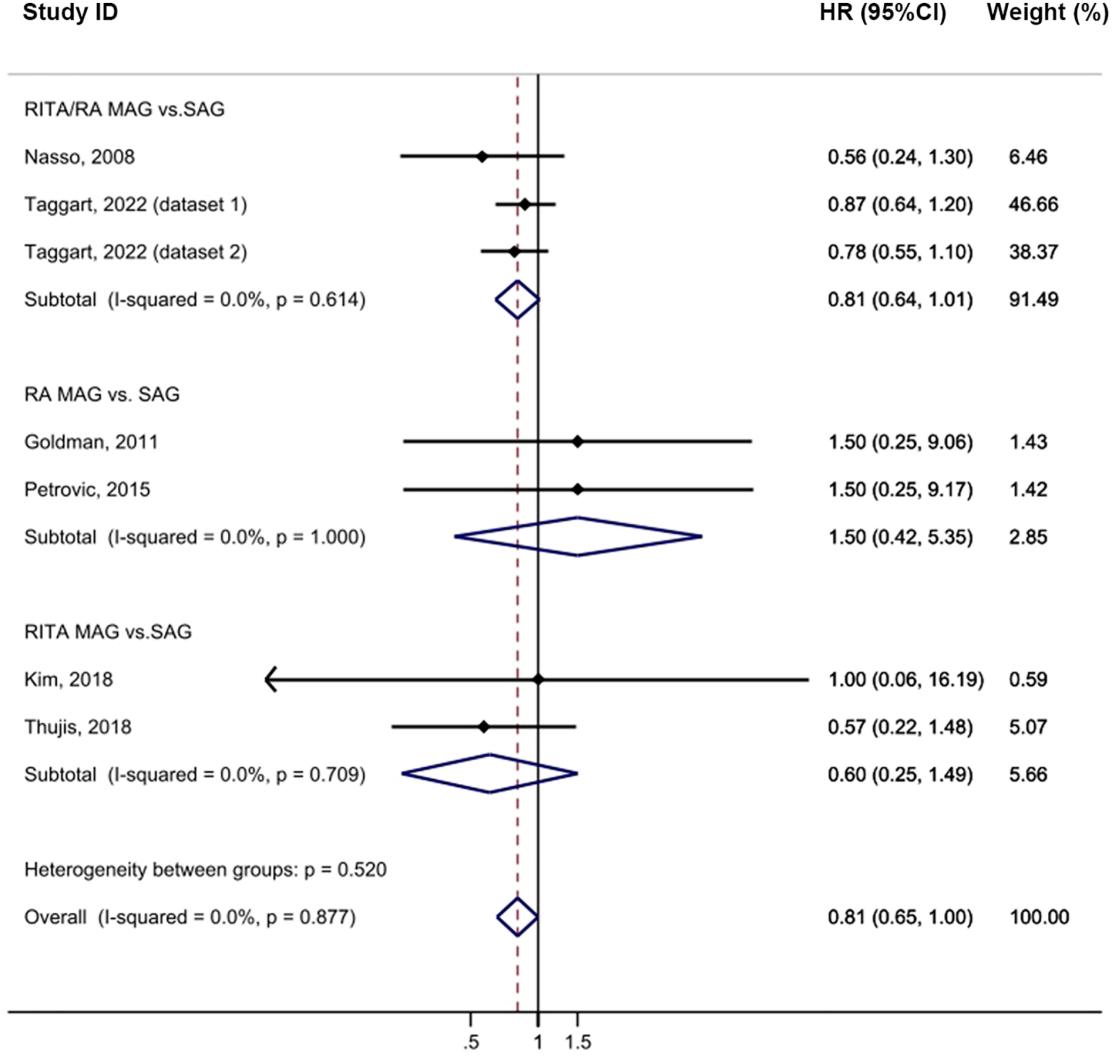
Forrest plot for cardiac mortality using intention-to-treat data.

Cardiac mortality was comparable between the MAG and SAG groups (HR = 0.81, 95% CI = 0.65–1.00, *p* = 0.05; Figure 3). No publication bias was evident in funnel plot analysis of these studies (Supplementary Figure S2B). Subgroup analyses revealed no significant differences between the established groups or across the different follow-up periods. Sensitivity analysis further showed that, upon excluding either the study by Goldman (17) or the study by Petrovic (14), the result became statistically significant (HR = 0.80, 95% CI = 0.64–0.99, *p* = 0.043; Supplementary Table S10).

### Secondary endpoints

**Figure 4:**
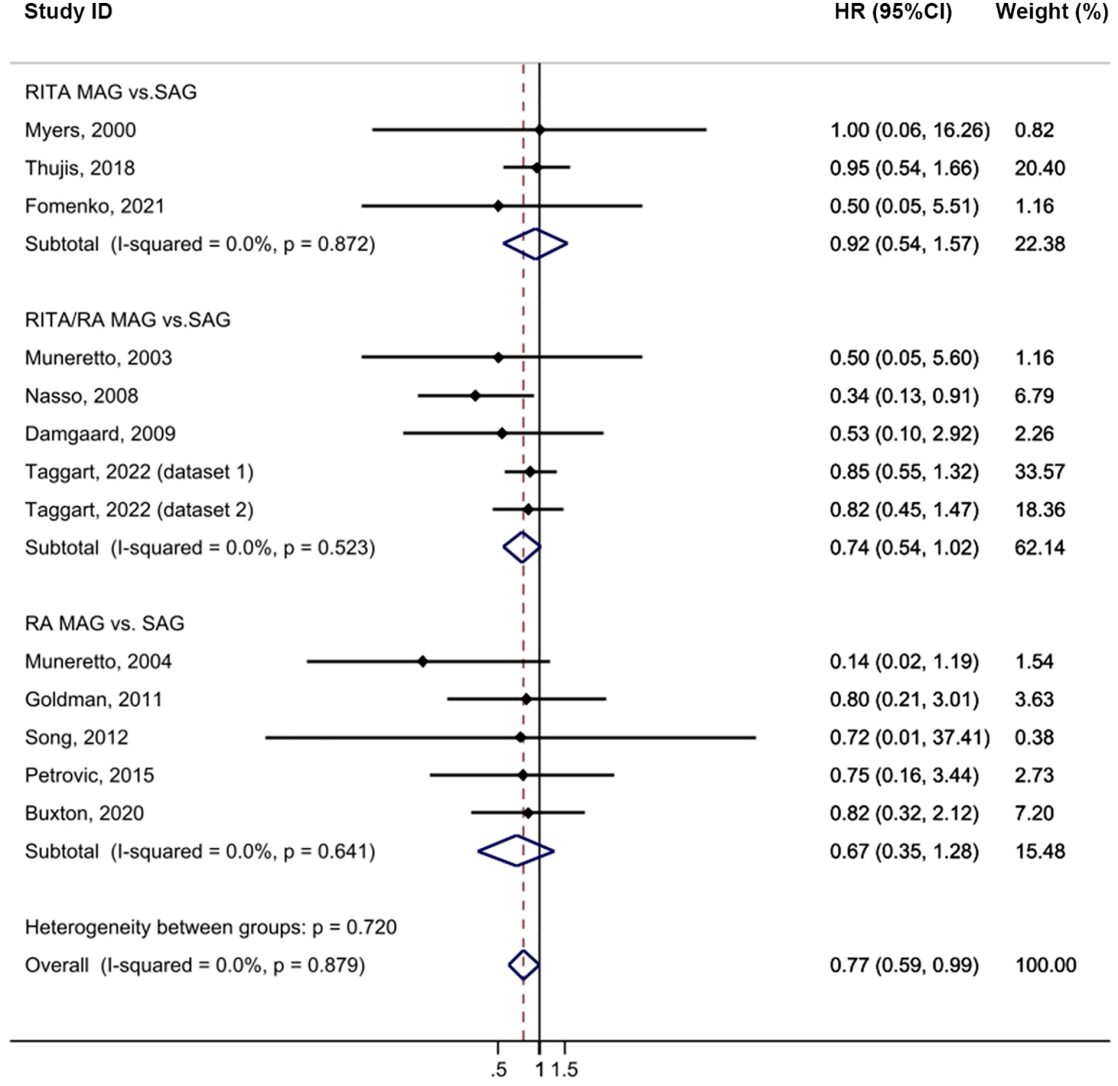
Forrest plot for myocardial infarction using intention-to-treat data.

Compared to the SAG groups, the MAG group demonstrated a lower incidence of MI after CABG, with no heterogeneity (HR = 0.77, 95% CI = 0.59–0.99, *p* = 0.039, I^2^ = 0%; Figure 4). No publication bias was evident in funnel plot analysis of these studies (Supplementary Figure S2C). Subgroup analysis revealed that this significant effect was primarily driven by the RITA/RA vs. SAG subgroups (HR = 0.74, 95% CI = 0.54–1.02, *p* = 0.067) and subgroups with a follow-up time of less than 5 years (HR = 0.67, 95% CI = 0.44–1.02, *p* = 0.061). Sensitivity analysis further showed that, upon excluding the study by Thuijs (25), the result became more statistically significant (HR = 0.72, 95% CI = 0.55–0.96, *p* = 0.026; Supplementary Table S10).

**Figure 5:**
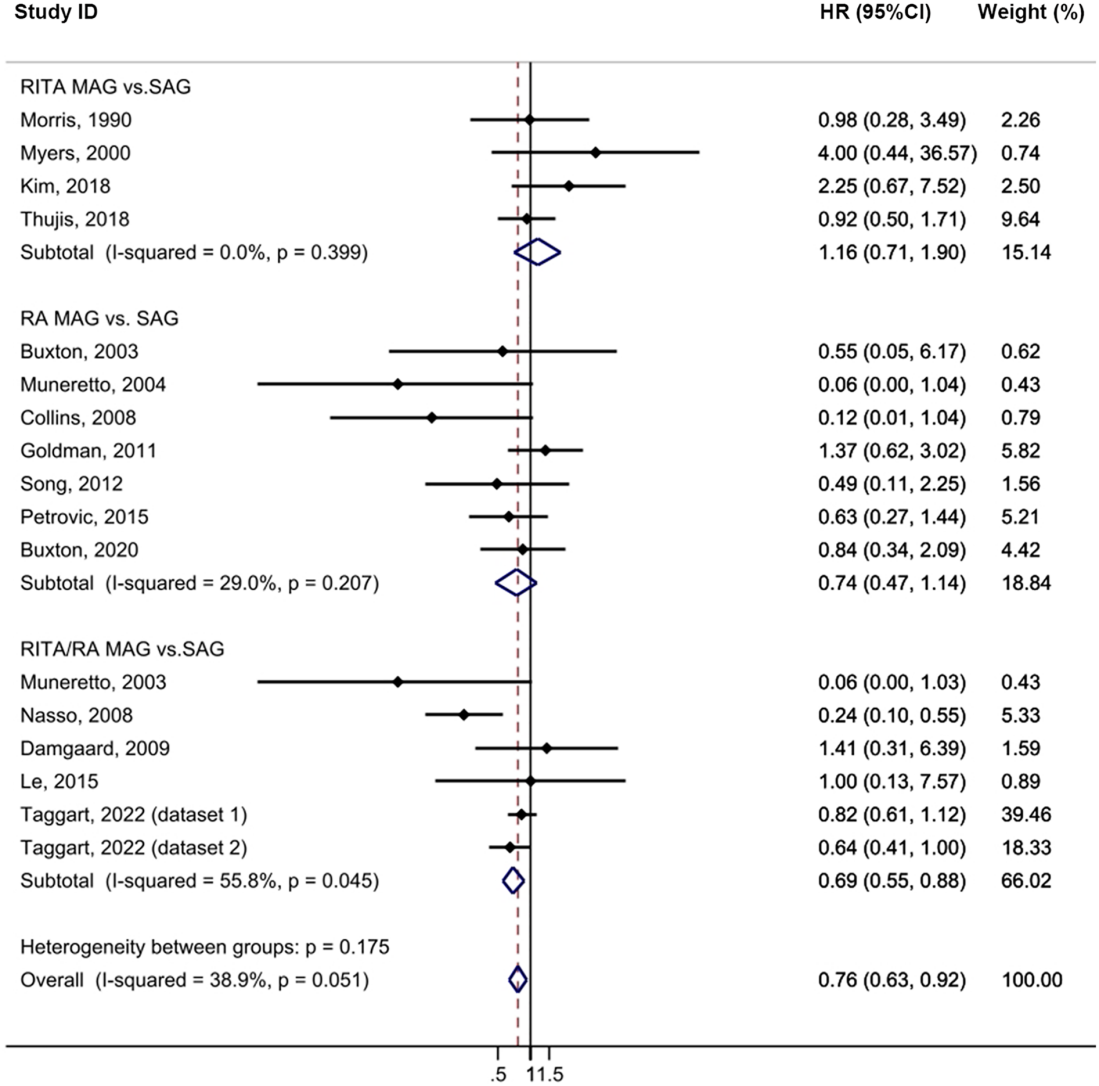
Forrest plot for repeat revascularization using intention-to-treat data.

The MAG group exhibited a statistically significant lower incidence of repeat revascularization compared to the SAG group with minimal heterogeneity (HR = 0.76, 95% CI = 0.63–0.92, *p* = 0.004, I^2^ = 38.9%; Figure 5). No publication bias was evident in funnel plot analysis (Supplementary Figure S2D). Subgroup analysis revealed that the RITA/RA MAG subgroup had a statistically significant lower incidence of this complication compared to the SAG group (HR = 0.69, 95% CI = 0.55–0.88, *p* = 0.002). Furthermore, this effect was pronounced in the subgroup with a follow-up time exceeding 10 years (HR = 0.76, 95% CI = 0.60–0.97, *p* = 0.029). In the sensitivity analysis, the exclusion of a single study did not cause the outcome to lose its statistical significance (Supplementary Table S10).

### Postoperative complications

There was no significant difference in the incidence of stroke between the MAG and SAG groups (HR = 0.83, 95% CI = 0.62–1.11, *p* = 0.214; Supplementary Figure S3). Similarly, the result of the subgroup analyses was not statistically significant. No publication bias was evident in funnel plot analysis (Supplementary Figure S2E). In the sensitivity analysis, the exclusion of any single study did not cause the outcome to be statistically significant (Supplementary Table S10).

**Figure 6:**
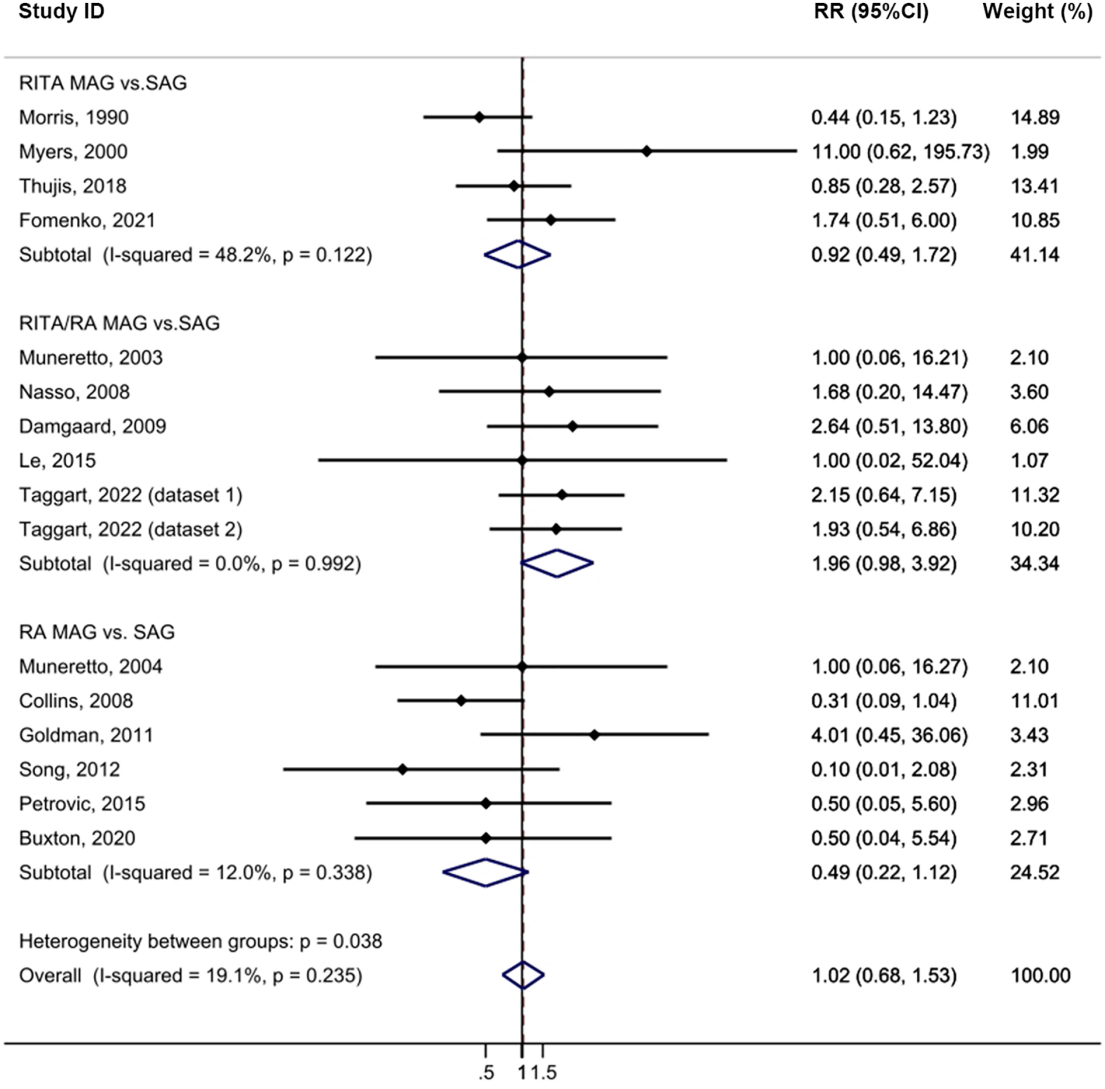
Forrest plot for sternal wound complications using intention-to-treat data.

The MAG and SAG groups did not demonstrate any statistically significant difference in the incidence of sternal wound complications following CABG with minimal heterogeneity (HR = 1.02, 95% CI = 0.68–1.53, *p* = 0.919, I^2^ = 19.1%; Figure 6). No publication bias was evident in the funnel plot analysis of the studies (Supplementary Figure S2F), and the results of the subgroup analysis were not statistically significant. Sensitivity analysis further confirmed that excluding any single study did not alter the statistical outcomes (Supplementary Table S10).

There was no statistically significant difference in the incidence of major bleeding between the MAG and SAG. However, the RA vs. SAG subgroup showed a reduced risk of bleeding with low heterogeneity (HR = 0.32, 95% CI = 0.10–0.90, *p* = 0.032, I^2^ = 35.2%; Supplementary Figure S4). No publication bias was evident in funnel plot analysis (Supplementary Figure S2G). Sensitivity analysis revealed that, upon excluding the study by Thuijs (25), the result became statistically significant (HR = 0.57, 95% CI = 0.32–0.99, *p* = 0.047; Supplementary Table S10).

The outcomes of the as-treated analysis were similar to those of the intention-to-treat analysis (Supplementary Tables S7, S8, and S11).

## Discussion

CABG, as an effective surgical treatment for coronary heart disease, has always been a key focus in clinical practice regarding the selection of vascular grafts. MAG and SAG, as two commonly used methods of vascular grafting, each have their unique advantages and disadvantages. This meta-analysis compared the outcomes of MAG and SAG, and showed that MAG was associated with higher survival from all-cause and cardiac mortality, lower risk of postoperative MI, and lower need for repeat revascularization, regardless of intention-to-treat or as-treated data. However, the absence of signiﬁcant differences were found in the postoperative outcomes such as sternal wound infection complications, major bleeding or stroke between MAG and SAG surgical revascularization.

Observational studies consistently favour MAG over SAG for multivessel coronary artery disease, showing better survival (8, 27–29). A study included over 1 million patients and found that MAG had a survival benefit over a 10-year follow-up period (30). This superiority could be explained by the greater patency rates of durable arterial grafts (31) or unmeasured confounders (32). Surprisingly the largest RCT addressing this issue to date, ART, had neutral outcomes (5). The possible explanation for this that an arterial graft (most commonly the LITA) is anastomosed to the most important vessel (commonly the LAD). Occlusion of a graft in a coronary artery other than the LAD artery may not have a survival effect (33).

Our findings are consistent with those of previous observational studies on mortality. In comparison with previous RCTs, our meta-analysis had a much larger sample size (5,047 MAG and 5,096 SAG). Our study included patients with RITA and RA MAG, whereas Gaudino et al. (534 with MAG, 502 with SAG) included only patients with RA (34). In addition, the follow-up period in our meta-analysis ranged from 0.5 to 12.6 years. A long follow-up period may yield a survival advantage in patients with MAG. This was evident in our subgroup analysis in which the benefits were pronounced in the subgroup with a follow-up period of >10 years. Sensitivity analysis showed that excluding the 1990 Morris study yielded statistically significant results, confirming this point. The MAG group showed a trend towards reduced cardiac mortality compared to the SAG group (*p* = 0.05). After excluding the studies by Goldman and Petrovic, the results were considered statistically significant. A potential reason could be the risk of competitive coronary ﬂow on arterial grafts. To minimise competitive flow, the RA is currently grafted to coronary arteries with stenosis of 90% or more of the vessel diameter (35). But Goldman et al. and Petrovic et al. used >70% and >80% proximal stenosis, respectively, as the entry criterion for a study vessel to receive an RA graft This could explain why there was no significant reduction in cardiac mortality in the MAG group.

MAG grafts have been shown to have superior angiographic patency compared to vein grafts (92% vs. 80% at 5 years) (34). Our result is consistent with the meta-analysis of Gaudino et al. (34), the risk of MI and repeat revascularization was lower with MAG than with SAG, in which RA MAG had lower MI (HR = 0.72, 95% CI = 0.53–0.99, *p* = 0.04) and decreased repeat revascularization rate (HR = 0.50, 95% CI = 0.40–0.63, *p* < 0.001) than that of SAG. The increased rates of postoperative MI and revascularization in the SAG group may be associated with the lower patency rate of the SVG. The average lifespan of an SVG is approximately ten years (36), and this effect becomes more pronounced as the follow-up period is extended.

MAG is not the primary approach in CABG because of the higher complexity of the surgical technique and concerns about postoperative SWIs. Francisca et al.'s study indicated that there was a nearly 2-fold increased risk of SWIs caused by MAG with BITA grafting compared with other grafting approaches whereas the RA grafting is not associated with an increased risk of SWIs (10). However, the *post hoc* analysis from the EXCEL trial reported neutral outcomes regarding the risk of SWIs caused by BITA vs. SITA grafting (25). The incidence of wound complications did not reach statistical significance in our study, although in most studies evaluating the rate of SWIs in the RITA and RITA/RA group, many of the confidence intervals cross one. This might be due to the improvements in surgical techniques and surgeons' understanding of skeletonized vessels (37, 38) have decreased the incidence of adverse events, including SWIs. The type of ITA was reported in four of 15 studies that evaluated SWIs (6, 12, 21, 23). Two studies used skeletonized harvesting methods (6, 12). The differences in vessel collection may be the reason for the insignificant effect size. Although we cannot determine the true direction of effect statistically, we need to pay attention to wound complications caused by MAG, and the use of skeletonized BITA can potentially reduce the incidence of SWIs.

Lower stroke rates in the MAG may be related to less aortic manipulation. A meta-analysis of observational studies conducted by Buttar et al. showed fewer cerebrovascular accidents with the RITA MAG than with the SAG (1.3% vs. 2.9%; *p* = 0.0003) (39). But the risk of stroke in our study was similar between the MAG and SAG groups. This finding may be attributed to the inclusion of a larger amount of RA data in our study. Furthermore, we found that the MAG did not increase the risk of major bleeding, particularly in the RA group, and that the risk of bleeding was significantly decreased. This may be related to characteristics such as the thicker arterial wall being less prone to injury and the arterial wall fitting snugly at the anastomotic site.

Although our research has demonstrated the survival benefits of MAG compared to SAG, there are still some limitations and uncertainties. Firstly, the studies included in our analysis vary in terms of trial design, sample size, and follow-up duration, which may lead to bias in the results. The ART study was the main contributor to the outcomes of this meta-analysis (weight of 39.61% for all-cause mortality) (11). However, the high crossover rates and the modification according to surgeon volume in the ART may influence the analysis of intention-to-treat data. To overcome the limitations of the ART, the ROMA trial (7), which is underway, will compare all MAG approaches and SAG without imposing on the surgeon which graft configuration should be adopted. Moreover, the EXCEL trial (25) and SYNTAX trail (3) included in our study were designed randomly to compare the effectiveness of PCI and CABG in patients with left main CAD, but SAG or MAG revascularization in the CABG group was chosen at the surgeon's discretion, and patients were not randomized, potentially leading to selection bias. For example, our meta-analysis revealed that MAG effectively reduced all-cause mortality (*p* = 0.004). Within the *post hoc* subgroup, MAG demonstrated a significant reduction in all-cause mortality (*p* = 0.012). However, in the subgroup of pure RCTs, MAG showed a trend towards decreasing all-cause mortality, but the results did not achieve statistical significance (*p* = 0.053). This outcome aligns with our expectation that the inclusion of *post hoc* analyses may have slightly inflated our effect size, because the *post hoc* analysis data is non-randomized in nature and may introduce selection bias to some extent. Consequently, future studies should adopt more stringent inclusion criteria and methodologies.

Except for Muneretto et al. did not report the patient randomization method (20), other studies (12, 14, 16) may have introduced selection bias by excluding high-risk patients with shorter life expectancies and those requiring emergency surgeries. In the study by Damgaard et al. (18), revascularization was performed by seven surgeons using different surgical techniques, which could lead to intervention bias. Furthermore, Kim et al. (13) found that eight patients in the SV group required a third limb conduit to lengthen the graft for complete revascularization, compared to 39 patients in the RITA group, and this difference could confound the outcomes. Muneretto included a high proportion of diabetic patients (40.5%) (21), and diabetes was an independent predictor of cardiac-related events and mortality, potentially leading to reporting bias. Thus, the selection criteria differed across studies and may have been based on the patients' clinical attributes and status, potentially leading to selection bias, and our results were not adjusted for biases.

Most RCTs that compared MAG and SAG were underpowered for long-term survival estimation, focused on angiographic primary outcomes, had small sample sizes (except for EXCEL and ART, all included less than 1,000 patients), and limited follow-up periods or rates. For instance, the CARRPO trial (18) and the study by Goldman et al. (17) estimated 5- year and 1-year graft patency, respectively, and both evaluated 1-year survival (3 and 16 deaths, respectively); the weight of these studies in this meta-analysis was 0.28% and 1.61%, respectively. Kim et al. (13) reported 5-year clinical outcomes but assessed 1-year angiographic patency. Song et al. (16) and Le et al. (15) estimated 1-year and 6-month graft patency, and the weight of these studies was 0.15% and 0.10%, respectively. The studies of Kim et al. (13), Damgaard et al. (18), and Le et al. (15) reported incomplete angiographic follow-up rates of 15%, 17%, and 24%, respectively. Missing data may introduce outcome bias.

Secondly, the results demonstrated the superiority in MAG compared to SAG but may have been affected by unmeasured confounders. For example, the EXCEL trail included perioperative MI (PMI) in the definition of major adverse cardiovascular and cerebrovascular events (MACCEs), garnering extensive controversy for its definition of MI and its powering (40, 41), in contrast to the NOBLE trial (42). The definition and detection of PMI varied across trials, limiting the interpretation of the results. The inclusion of PMI may overestimate the incidence of MI, as minor myocardial injuries detected in routine tests would be classified as MI, although these injuries are not necessarily clinically significant adverse events. Furthermore, the design of the EXCEL trial may tend to demonstrate the superiority of CABG, especially in long-term follow-up (43). These differences in trial design may have influenced the interpretation of the results.

Either BITA (8) or SITA+RA (44) grafting has a survival benefit over SITA grafting. Due to the lack of clear evidence, the potentially increased sternal wound complication rate, and the perceived technical complexity when using bilateral internal thoracic arteries often results in the RA as the preferred second conduit of choice. The proportion of patients receiving BITA grafts tends to decrease as the RA becomes more frequently utilized as the second conduit, although BITA grafts have better long-term patency and survival rates (45). The effect size of the subgroup of patients receiving RA/RITA grafting in our study was insignificant. This result may be attributed to the widespread use of the RA in this subgroup (data not provided), limiting the potential benefits of BITA grafts. Moreover, Lytle et al. suggested that sufficient follow-up time is needed to clarify the unequivocal long-term survival beneﬁts of BITA (46).

Thirdly, our study failed to fully evaluate the differences in response to MAG and SAG among specific patient subgroups, which limits the generalizability of our findings. A higher mortality in females who have undergone CABG compared to males has been well-documented in many studies (47, 48). Female sex is an independent predictor of operative and long-term mortality after CABG (48). This phenomenon may be related to sex differences in biology and baseline risk characteristics, as well as smaller coronary artery diameter and body surface area in women (49). In addition, some widely recognized risk factors include older age, poorer renal function, lower BMI, and the comorbidity of diabetes mellitus, hypertension, peripheral arterial disease, or COPD (50, 51). Observational studies focused on the comparison between MAG and SAG, which patients receiving MAG had a low percentage of females, and fewer comorbidities, including diabetes, peripheral vascular disease, and extensive CAD etc. (8). These factors could explain the better outcomes of MAG. However, in our study, the sex distribution and the proportion of preoperative complications such as hypertension, diabetes mellitus, previous MI and COPD was similar between the two groups. The MAG group has a lower prevalence of obesity and peripheral arterial disease, but a higher incidence of hyper lipidaemia. Although the included studies provide limited data, we must acknowledge that these factors may confound the results. Thus, future studies should perform subgroup analyses of MAG outcomes by sex, complications, and other variables to determine the patient groups more likely to benefit from MAG.

In conclusion, compared with SAG, MAG was associated with higher survival, lower risk of MI, and lower need for repeat revascularization, and the benefits increasing as the follow-up time prolongs. Nonetheless, there were no significant differences in the incidence of sternal wound infections, major bleeding, and stroke between MAG and SAG.

In the future, additional meta-analyses are necessary to investigate the impact of baseline characteristics, including sex, BMI and comorbidities etc, on the choice of conduit; and independently analysis are needed regarding non-survival-based outcomes to comprehensively understand the safety of MAG. Therefore, we recommend that the decision to use MAG or SAG should be individualised.

## Methods

According to the Preferred Reporting Items for Systematic Reviews and Meta-Analyses guidelines (Supplementary Table S1) (52, 53), we conducted a systematic search of PubMed, Google, and Web of Science databases from inception until December 2024 with English language without geographical restrictions. We defined MeSH terms and free text terms to define each component of the PICO expression: P) Population, coronary artery disease adult patients submit ted to CABG procedure; I) Intervention, multiple arterial grafts; C) Comparison, single arterial graft and O) Outcomes, death, survival, MACCEs, MI, repeat revascularization, and postoperative complications (SWIs, stroke, and bleeding). The search queries are shown in Supplementary Tables S2–S4.

Duplicates, reviews, meta-analysis, cross-sectional and case-control studies, case series, case reports, abstracts conference presentations, editorials and expert opinions, and studies that did not report HRs or RRs were excluded. The inclusion criteria were (1) studies on adults (age ≥18 years), (2) studies published in English, (3) RCTs or *post hoc* analyses, (4) studies that evaluated the outcomes of MAG and SAG; and 5) studies with follow-up periods of at least 6 months. The flowchart of study selection is shown in Figure 1.

Data extraction was independently conducted by two authors (Q.D. and Q.Z.), and any discrepancies were resolved through discussion and adjudication by a senior author (M.G.). The demographic data and study characteristics were recorded. The assessed clinical outcomes included primary endpoints: all-cause mortality, cardiac mortality; secondary endpoints: MI, repeat revascularization; and postoperative complications: stroke, sternal wound complications, major bleeding. The outcomes were evaluated during the maximum follow-up period.

The studies were divided into three subgroups based on the arterial grafts used in the MAG group: (1) RITA MAG: The first arterial graft was LITA and second arterial graft used was RITA. (2) RA MAG: The first and second arterial grafts were the LITA and RA, respectively. (3) RITA/RA MAG: the first arterial graft was the LITA, and the second arterial graft was either the RITA or RA. All SVGs were used in addition to and after the arterial grafts were used. The SAG group consistently had one arterial graft in the LAD and additional venous grafts in the other coronary vessels.

The RCT quality was assessed using the Revised Cochrane Risk-of-Bias Tool for Randomised Trials (RoB2) (54). The pooled HR or RR was calculated for the outcomes using the generic inverse variance method. Tests of heterogeneity of the HRs across studies were estimated using the chi-square and I-square (I^2^) tests, with fixed-effects or random-effects models based on the criteria of *P* > 0.10 and I^2^ < 50%. Funnel plots were used to assess publication bias, and sensitivity analysis was applied to measure the influence of each study. Statistical significance was defined as a two-tailed *P*-value <0.05. All data were analysed using Stata (version 17.0; Stata Corp., College Station, TX, USA).

## Data Availability

The original contributions presented in the study are included in the article/Supplementary Material, further inquiries can be directed to the corresponding authors.
