# Supplementary material for: Comparison of multiple arterial grafts vs. single arterial graft in coronary artery bypass surgery: a systematic review and meta-analysis
Source: Front Cardiovasc Med. 2025 Mar 27;12:1555242. doi: 10.3389/fcvm.2025.1555242 (PMC11983608; doi:10.3389/fcvm.2025.1555242)
Supplement: Supplementary file 1 [file Datasheet1.pdf]

Comparison of multiple arterial grafts vs. single arterial graft in coronary artery bypass surgery: A systematic review and meta-analysis

Qiuju Ding[#1], Qingqing Zhu[#1], Lichong Lu[1], Xiaofeng Cheng[*1.2], Min Ge[*1]

[1]Department of Cardio-thoracic Surgery, Nanjing Drum Tower Hospital, The Affiliated Hospital of Nanjing University Medical School, Nanjing, China

[2]Department of Cardio-Thoracic Surgery, Nanjing Drum Tower Hospital, Clinical College of Nanjing University of Chinese Medicine, Nanjing, China.

[#]These authors contributed equally.

***Corresponding author:**

Xiaofeng Cheng, Ph.D., M.D.

Department of Cardio-thoracic Surgery, Nanjing Drum Tower Hospital, The Affiliated Hospital of Nanjing University Medical School, Zhongshan Road 215, Nanjing 210008, China.

E-mail: chengxiaofeng_glyy@163.com

Min Ge, Ph.D., M.D.

Department of Cardio-thoracic Surgery, Nanjing Drum Tower Hospital, The Affiliated Hospital of Nanjing University Medical School, Zhongshan Road 321, Nanjing 210008, China.

Email: gemin2000@outlook.com

Supplementary Figure S1. Risk of bias within randomized controlled trials (Cochrane Risk of Bias).

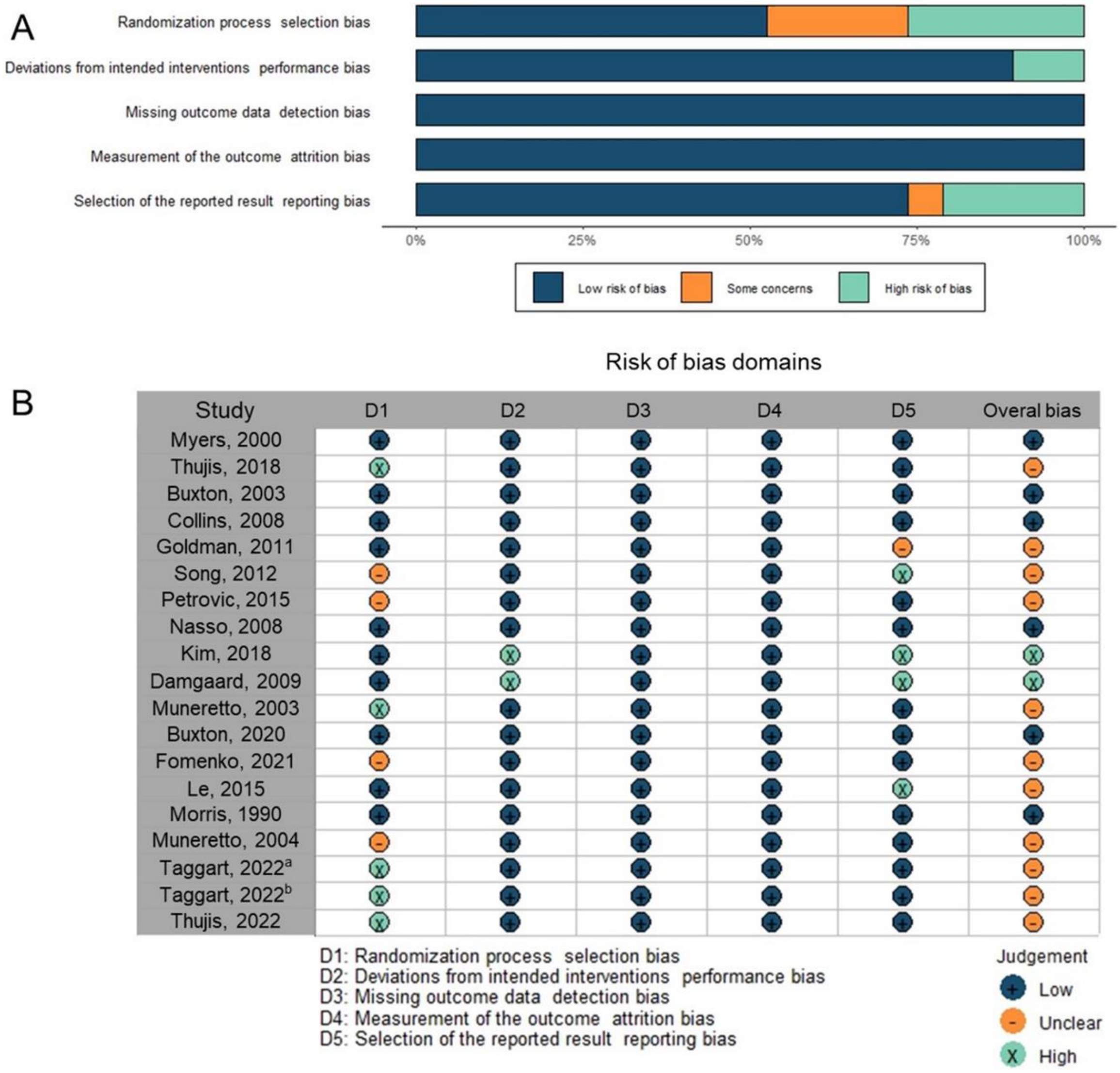

Supplementary Figure S2. Funnel plot for (A) all-cause mortality, (B) cardiac mortality, (C) myocardial infarction, (D) repeat revascularization, (E) stroke, (F) sternal wound complications and (G) bleeding complications.

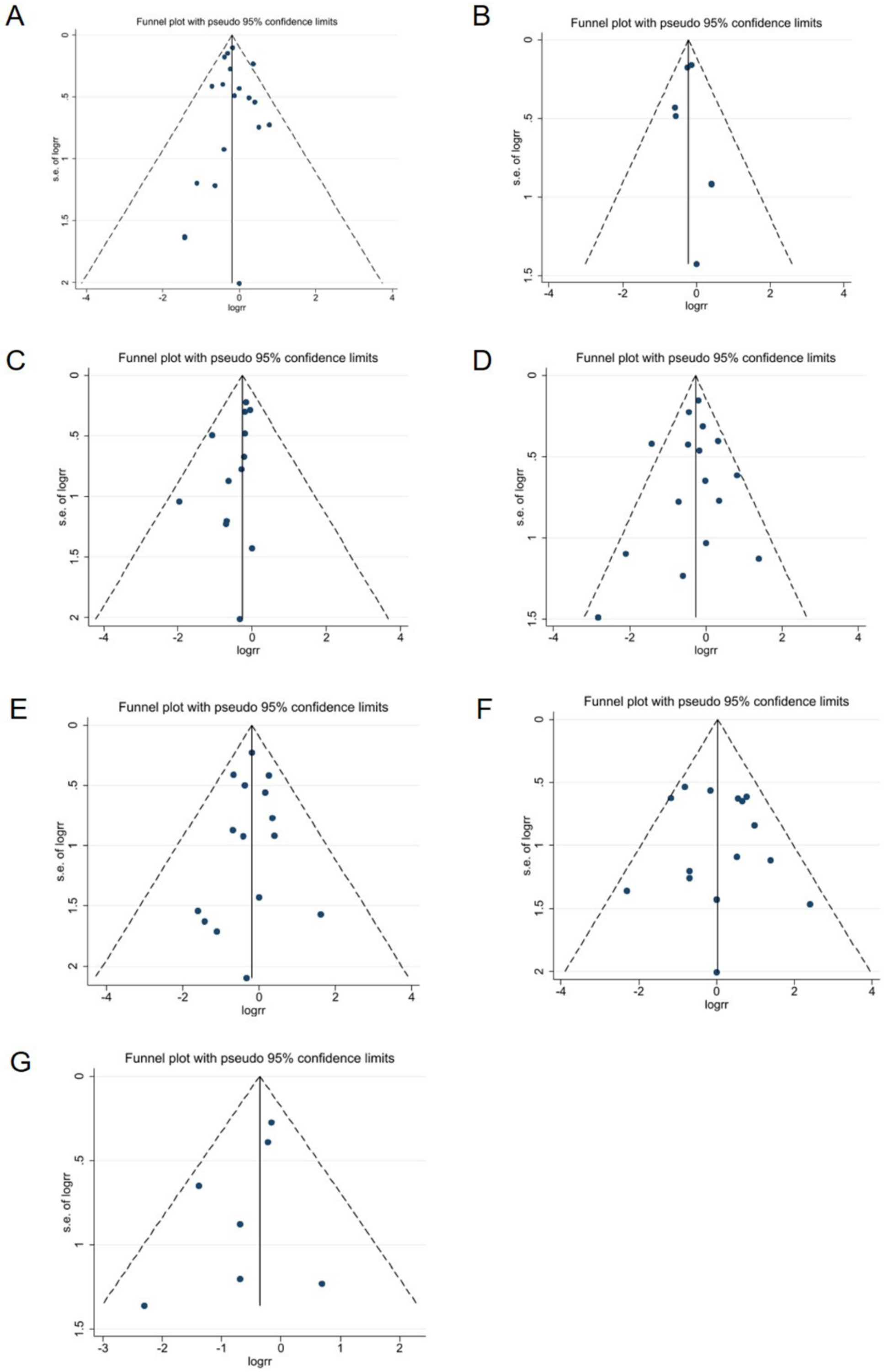

Supplementary Figure S3. Forrest plot for stroke using intention to treat data.

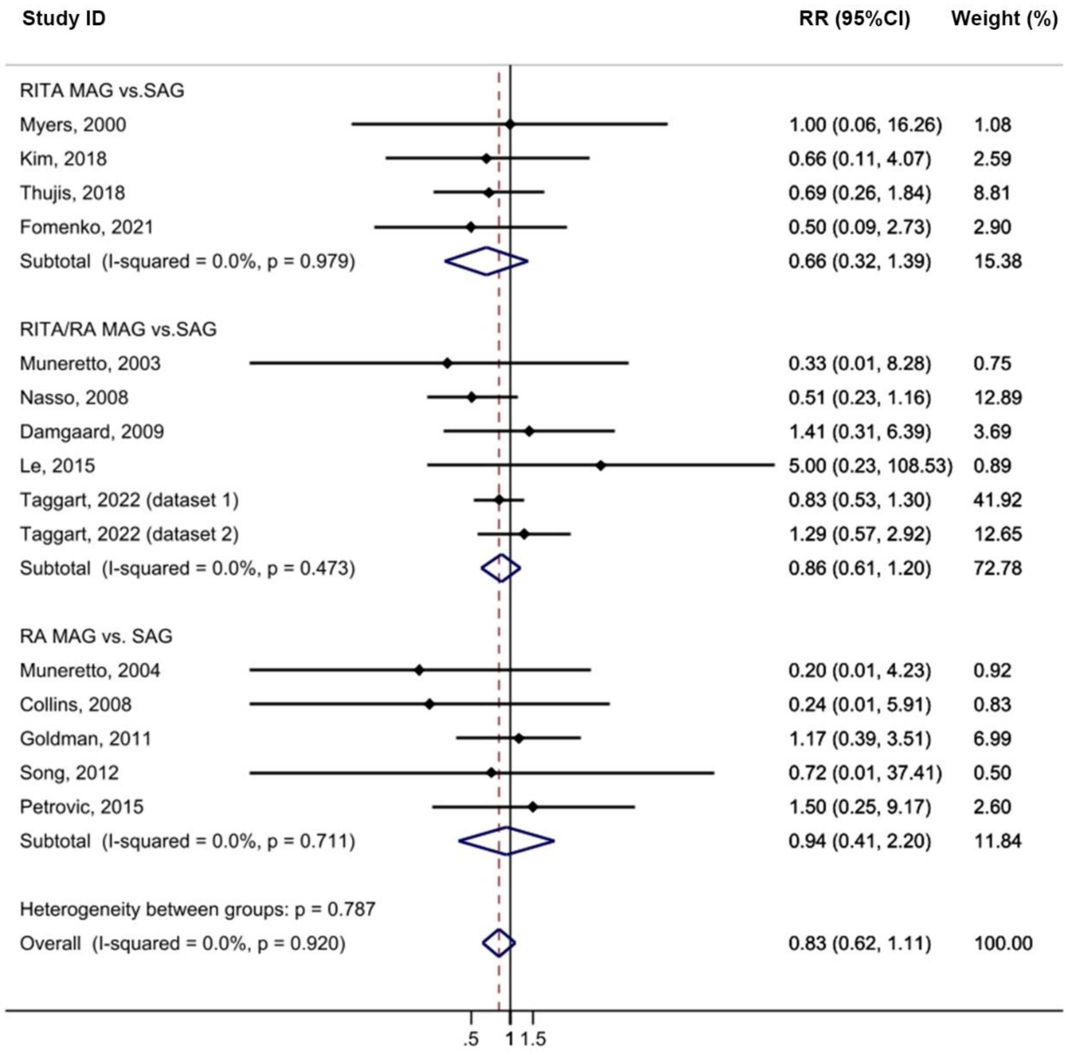

Supplementary Figure S4. Forrest plot for bleeding complications using intention to treat data.

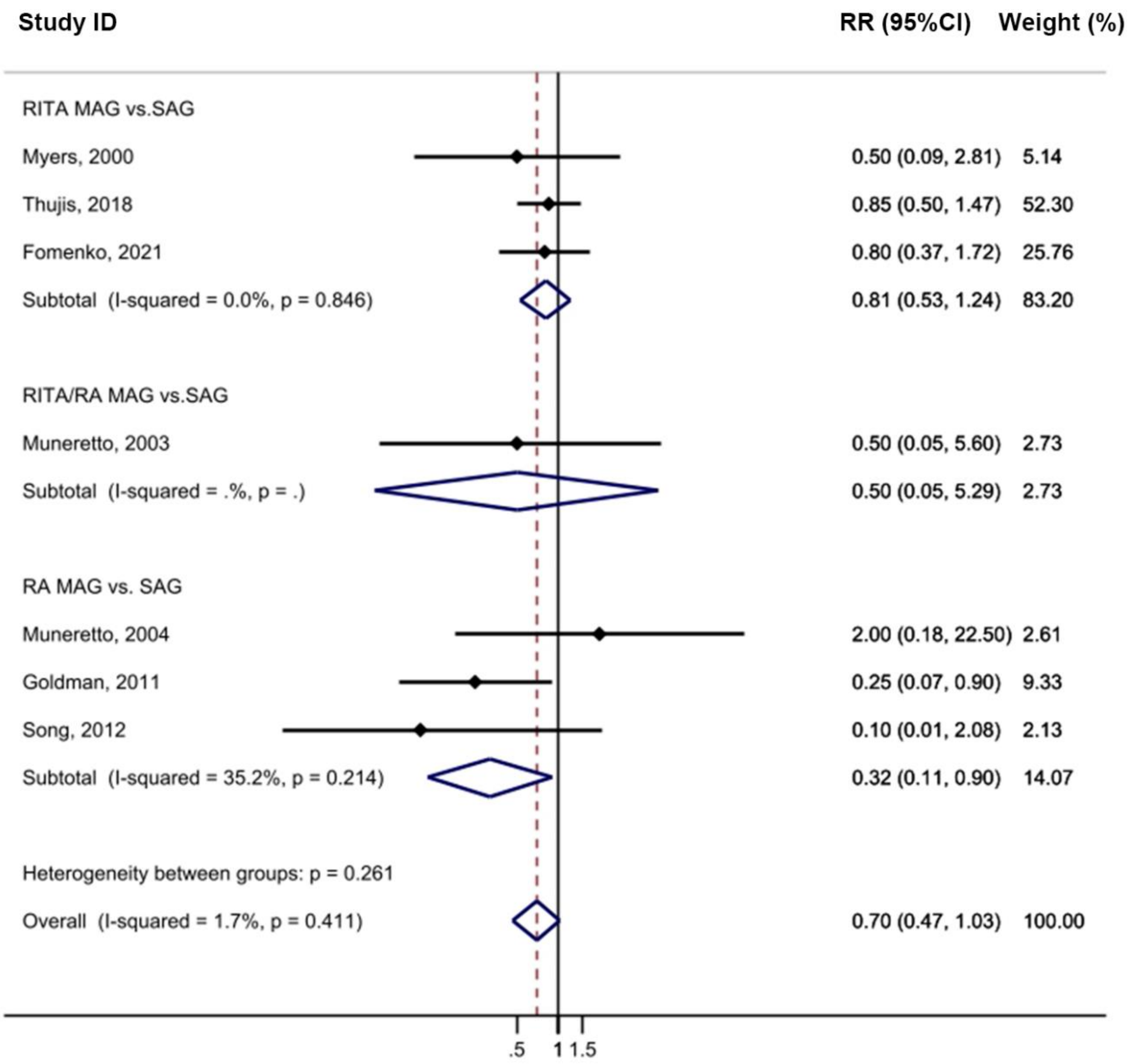

Supplementary Table S1. PRISMA 2020 checklist

Section and Topic	Item #	Checklist item	Location where item is reported
**TITLE**			
Title	1	Identify the report as a systematic review.	Title page
**ABSTRACT**			
Abstract	2	See the PRISMA 2020 for Abstracts checklist.	Abstract page
**INTRODUCTION**			
Rationale	3	Describe the rationale for the review in the context of existing knowledge.	Introduction page
Objectives	4	Provide an explicit statement of the objective(s) or question(s) the review addresses.	Introduction page
**METHODS**			
Eligibility criteria	5	Specify the inclusion and exclusion criteria for the review and how studies were grouped for the syntheses.	Methods page, Figure 1
Information sources	6	Specify all databases, registers, websites, organisations, reference lists and other sources searched or consulted to identify studies. Specify the date when each source was last searched or consulted.	Methods page, Figure 1, Supplementary Table 2-4
Search strategy	7	Present the full search strategies for all databases, registers and websites, including any filters and limits used.	Figure 1, Supplementary Table 2-4
Selection process	8	Specify the methods used to decide whether a study met the inclusion criteria of the review, including how many reviewers screened each record and each report retrieved, whether they worked independently, and if applicable, details of automation tools used in the process.	Methods page
Data collection process	9	Specify the methods used to collect data from reports, including how many reviewers collected data from each report, whether they worked independently, any processes for obtaining or confirming data from study investigators, and if applicable, details of automation tools used in the process.	Methods page
Data items	10a	List and define all outcomes for which data were sought. Specify whether all results that were compatible with each outcome domain in each study were sought (e.g. for all measures, time points, analyses), and if not, the methods used to decide which results to collect.	Methods page, Table 1, Supplementary Table 5
	10b	List and define all other variables for which data were sought (e.g. participant and	Methods page, Table 1

Section and Topic	Item #	Checklist item	Location where item is reported
		intervention characteristics, funding sources). Describe any assumptions made about any missing or unclear information.	Partially Not Applicable
Study risk of bias assessment	11	Specify the methods used to assess risk of bias in the included studies, including details of the tool(s) used, how many reviewers assessed each study and whether they worked independently, and if applicable, details of automation tools used in the process.	Methods page, Supplementary Figure 1
Effect measures	12	Specify for each outcome the effect measure(s) (e.g. risk ratio, mean difference) used in the synthesis or presentation of results.	Methods page
Synthesis methods	13a	Describe the processes used to decide which studies were eligible for each synthesis (e.g. tabulating the study intervention characteristics and comparing against the planned groups for each synthesis (item #5)).	Methods page, Figure 1
	13b	Describe any methods required to prepare the data for presentation or synthesis, such as handling of missing summary statistics, or data conversions.	Methods page
	13c	Describe any methods used to tabulate or visually display results of individual studies and syntheses.	Methods page
	13d	Describe any methods used to synthesize results and provide a rationale for the choice(s). If meta-analysis was performed, describe the model(s), method(s) to identify the presence and extent of statistical heterogeneity, and software package(s) used.	Methods page
	13e	Describe any methods used to explore possible causes of heterogeneity among study results (e.g. subgroup analysis, meta-regression).	Methods page
	13f	Describe any sensitivity analyses conducted to assess robustness of the synthesized results.	Methods page
Reporting bias assessment	14	Describe any methods used to assess risk of bias due to missing results in a synthesis (arising from reporting biases).	Methods page
Certainty assessment	15	Describe any methods used to assess certainty (or confidence) in the body of evidence for an outcome.	Methods page
**RESULTS**			
Study selection	16a	Describe the results of the search and selection process, from the number of records identified in the search to the number of studies included in the review, ideally using a flow diagram.	Results page, Figure 1
	16b	Cite studies that might appear to meet the inclusion criteria, but which were excluded,	Results page, Figure 1

Section and Topic	Item #	Checklist item	Location where item is reported
		and explain why they were excluded.	
Study characteristics	17	Cite each included study and present its characteristics.	Results page, Table 1
Risk of bias in studies	18	Present assessments of risk of bias for each included study.	Supplementary Figure 1
Results of individual studies	19	For all outcomes, present, for each study: (a) summary statistics for each group (where appropriate) and (b) an effect estimate and its precision (e.g. confidence/credible interval), ideally using structured tables or plots.	Results page, Figure 2-6, Supplementary Figure 3-4, Table S6-S10
Results of syntheses	20a	For each synthesis, briefly summarise the characteristics and risk of bias among contributing studies.	Supplementary Figure 1
	20b	Present results of all statistical syntheses conducted. If meta-analysis was done, present for each the summary estimate and its precision (e.g. confidence/credible interval) and measures of statistical heterogeneity. If comparing groups, describe the direction of the effect.	Results page, Figure 2-6, Supplementary Figure 3-4, Table S6-S10
	20c	Present results of all investigations of possible causes of heterogeneity among study results.	Results page, Table S9-S10
	20d	Present results of all sensitivity analyses conducted to assess the robustness of the synthesized results.	Results page, Table S9-S10
Reporting biases	21	Present assessments of risk of bias due to missing results (arising from reporting biases) for each synthesis assessed.	Not applicable
Certainty of evidence	22	Present assessments of certainty (or confidence) in the body of evidence for each outcome assessed.	Results page
**DISCUSSION**			
Discussion	23a	Provide a general interpretation of the results in the context of other evidence.	Discussion page
	23b	Discuss any limitations of the evidence included in the review.	Discussion page
	23c	Discuss any limitations of the review processes used	Discussion page
	23d	Discuss implications of the results for practice, policy, and future research.	Discussion page
**OTHER INFORMATION**			
Registration and protocol	24a	Provide registration information for the review, including register name and registration number, or state that the review was not registered.	Not registered

Section and Topic	Item #	Checklist item	Location where item is reported
	24b	Indicate where the review protocol can be accessed, or state that a protocol was not prepared.	Not prepared
	24c	Describe and explain any amendments to information provided at registration or in the protocol.	Not applicable
Support	25	Describe sources of financial or non-financial support for the review, and the role of the funders or sponsors in the review.	See Acknowledgements
Competing interests	26	Declare any competing interests of review authors.	See Competing Interests Statement
Availability of data, code and other materials	27	Report which of the following are publicly available and where they can be found: template data collection forms; data extracted from included studies; data used for all analyses; analytic code; any other materials used in the review.	See Data availability statement

Supplementary Table S2. Medline Search Strategy

Population	#1	“vein graft” OR “vein grafting” OR “arterial graft” OR “radial artery graft” OR “radial artery grafting” OR “radial arteries graft” OR “radial arteries grafting “arterial grafting” OR “vein grafting” OR “internal mammary artery graft” OR “internal mammary artery grafting” OR “internal thoracic artery grafting” OR “internal thoracic artery graft” [Mesh] OR "Mammary Arteries"[Mesh] OR "Internal Mammary-Coronary Artery Anastomosis"[Mesh] OR "radial Artery/transplantation"[Mesh] OR "coronary bypass" OR “coronary bypass graft” OR “coronary bypass grafting” OR “coronary artery bypass” OR “coronary arteries bypass” OR "coronary artery bypass graft" OR "coronary artery bypass grafting" OR “coronary arteries bypass graft” OR “coronary arteries bypass grafting” OR "cardiac bypass" OR "coronary heart surgery" OR “cabg” OR “coronary revascularization” OR “cardiac revascularization” OR “myocardial revascularization” OR “Coronary Artery Bypass"[Mesh] OR "Coronary Artery Disease/surgery"[Mesh] OR "Coronary Artery Bypass, Off-Pump"[Mesh] OR "Myocardial Revascularization"[Mesh] OR "Myocardial Ischemia/surgery"[Mesh] OR "Coronary Disease/surgery"[Mesh] OR "Vascular Grafting"[Mesh] OR "Coronary Vessels/surgery"[Mesh]
Intervention	#2	“radial graft” OR “multiple arterial graft” OR “multiple arterial grafting” OR “bilateral internal thoracic artery” OR “bilateral internal thoracic arteries” OR “bilateral internal mammary arteries” OR “bilateral internal mammary artery” OR “double internal thoracic artery” OR “double internal thoracic arteries” OR “double internal mammary artery” OR “double internal mammary arteries” OR “total arterial” OR “multiple arterial” OR “multiple arterial reconstructions” OR “multiple arterial revascularization” OR “multiple arterial revascularizations” OR “bima” OR “bita”
Comparison	#3	“radial graft” OR “single arterial graft” OR “single arterial grafting” OR “single internal thoracic artery” OR “single internal thoracic arterial” OR “single internal mammary artery” OR “single internal mammary arterial” OR “unilateral internal thoracic artery” OR “unilateral internal thoracic arterial” OR “unilateral internal mammary artery” OR “unilateral internal mammary arterial” OR “internal mammary artery graft” OR “internal mammary artery grafting” OR “internal thoracic artery grafting” OR “internal thoracic artery graft” OR “sima” OR “sita”

Outcomes	#4	“Mortality” OR “Survival” OR “Death” OR “survival rate” OR “cumulative survival” OR “death rate” OR “all-causes mortality” OR “in-hospital death” OR “early death” OR “hospital mortality” OR “30-days mortality” OR “hospital death” OR “in-hospital mortality” OR “major adverse cardiac and cerebrovascular events” OR “adverse cardiac events” OR “adverse cardiac event” OR “adverse cerebrovascular event” OR “adverse cerebrovascular events” OR “adverse cerebral events” OR “macce” OR “macces” OR “myocardial infarct” OR “myocardial infarction” OR “myocardial ischemia” OR “heart attack” OR “acute coronary syndrome” OR “stroke” OR “acute coronary syndromes” OR “cerebrovascular accident” OR “cerebrovascular accidents” OR “cva” OR “cerebral accident” OR “cerebral accidents” OR "Mortality"[Mesh] OR "Survival"[Mesh] OR "Death"[Mesh] OR "Survival Rate"[Mesh] OR "Survival Analysis"[Mesh] OR "Cause of Death"[Mesh] OR "Hospital Mortality"[Mesh] OR "Cardiovascular Diseases/mortality"[Mesh] OR "Myocardial Ischemia/mortality"[Mesh] OR "Myocardial Infarction/mortality"[Mesh] OR "Myocardial Infarction"[Mesh] OR "Myocardial Ischemia"[Mesh] OR "Acute Coronary Syndrome"[Mesh] OR "Stroke"[Mesh] OR "repeat revascularization"[Mesh]OR "graft narrowing "[Mesh]OR "sternal wound complications"[Mesh]OR "sternal wound infection"[Mesh] "bleeding"[Mesh]OR "graft patency"[Mesh]
Language	#5	"English"[Language]
Date of publication	#6	"1960/01/01"[Date - Publication] : "2024/12/31"[Date - Publication]
Excluded studies	#7	"case reports"[Publication Type] OR "meta-analysis"[Publication Type] OR "review"[Publication Type] OR “clinical trial” [Publication Type]
RCT	#8	“randomized controlled trial"[Publication Type]
Final		#1 AND #2 AND #3 AND #4 AND #5 AND #6 NOT #7 AND #8

Supplementary Table S3. Web of science search strategy

Population	#1	TS = ((vein and graft*) OR (arterial and graft*) OR (radial and arter* and graft*) OR (internal mammary and arter* and graft*) OR (internal thoracic and arter* and graft*) OR (coronary and bypass*) OR (coronary bypass and graft*) OR (coronary arter* and bypass) OR (coronary arter* and bypass graft*) OR "cardiac bypass" OR (coronary heart and surger*) OR cabg OR (coronary and revascularization*) OR (cardiac and revascularization*) OR (myocardial and revascularization*))
Intervention	#2	TS = ((radial and graft*) OR (multiple arterial and graft*) OR (bilateral internal thoracic and arter*) OR (bilateral internal mammary and arter*) OR (double internal thoracic and arter*) OR (double internal mammary and arter*) OR "total arterial" OR "multiple arterial" OR (multiple arterial and reconstruction*) OR (multiple arterial and revascularization*) OR bima OR bita)
Comparison	#3	TS = ((radial and graft*) OR (single arterial and graft*) OR (single internal thoracic and arter*) OR (single internal mammary and arter*) OR (unilateral internal thoracic and arter*) OR (unilateral internal mammary and arter*) OR (internal mammary artery and graft*) OR (internal thoracic artery and graft*) OR sima OR sita)
Outcomes	#4	TS = (“Mortality” OR “Survival” OR “Death” OR “survival rate” OR “cumulative survival” OR “death rate” OR “all-causes mortality” OR “in-hospital death” OR “early death” OR “hospital mortality” OR “30-days mortality” OR “hospital death” OR “in-hospital mortality” OR “major adverse cardiac” and “cerebrovascular events” OR “adverse cardiac events” OR “adverse cardiac event” OR “adverse cerebrovascular event” OR “adverse cerebrovascular events” OR “adverse cerebral events” OR “macce” OR “macces” OR “myocardial infarct” OR “myocardial infarction” OR “myocardial ischemia” OR “heart attack” OR “acute coronary syndrome” OR “stroke” OR “acute coronary syndromes” OR “cerebrovascular accident” OR “cerebrovascular accidents” OR “cva” OR “cerebral accident” OR “cerebral accidents" OR "repeat revascularization" OR "graft narrowing "OR "sternal wound complications" OR "sternal wound infection" OR "bleeding" OR "graft patency")
Language	#5	IDIOMA: (English)

Date of publication	#6	Time span=1900-2024
RCT	#7	TS = ((randomized controlled trial) OR (randomized controlled trials) OR (clinical trial) OR (clinical trials) OR (controlled clinical trial) OR (randomised controlled trial) OR (randomised controlled trials))
Final		#1 AND #2 AND #3 AND #4 AND #5 AND #6 AND #7

Supplementary Table S4. Google search strategy

Population	#1	((vein and graft*) OR (arterial and graft*) OR (radial and arter* and graft*) OR (internal mammary and arter* and graft*) OR (internal thoracic and arter* and graft*) OR (coronary and bypass*) OR (coronary bypass and graft*) OR (coronary arter* and bypass) OR (coronary arter* and bypass graft*) OR "cardiac bypass" OR (coronary heart and surger*) OR cabg OR (coronary and revascularization*) OR (cardiac and revascularization*) OR (myocardial and revascularization*))
Intervention	#2	((radial and graft*) OR (multiple arterial and graft*) OR (bilateral internal thoracic and arter*) OR (bilateral internal mammary and arter*) OR (double internal thoracic and arter*) OR (double internal mammary and arter*) OR "total arterial" OR "multiple arterial" OR (multiple arterial and reconstruction*) OR (multiple arterial and revascularization*) OR bima OR bita)
Comparison	#3	((radial and graft*) OR (single arterial and graft*) OR (single internal thoracic and arter*) OR (single internal mammary and arter*) OR (unilateral internal thoracic and arter*) OR (unilateral internal mammary and arter*) OR (internal mammary artery and graft*) OR (internal thoracic artery and graft*) OR sima OR sita)
Outcomes	#4	(“Mortality” OR “Survival” OR “Death” OR “survival rate” OR “cumulative survival” OR “death rate” OR “all-causes mortality” OR “in-hospital death” OR “early death” OR “hospital mortality” OR “30-days mortality” OR “hospital death” OR “in-hospital mortality” OR “major adverse cardiac” and “cerebrovascular events” OR “adverse cardiac events” OR “adverse cardiac event” OR “adverse cerebrovascular event” OR “adverse cerebrovascular events” OR “adverse cerebral events” OR “macce” OR “macces” OR “myocardial infarct” OR “myocardial infarction” OR “myocardial ischemia” OR “heart attack” OR “acute coronary syndrome” OR “stroke” OR “acute coronary syndromes” OR “cerebrovascular accident” OR “cerebrovascular accidents” OR “cva” OR “cerebral accident” OR “cerebral accidents" OR "repeat revascularization" OR "graft narrowing "OR "sternal wound complications" OR "sternal wound infection" OR "bleeding" OR "graft patency")
Language	#5	English
Date of publication	#6	before 2024

RCT	#7	((randomized controlled trial) OR (randomized controlled trials) OR (clinical trial) OR (clinical trials) OR (controlled clinical trial) OR (randomised controlled trial) OR (randomised controlled trials))
Final		#1 AND #2 AND #3 AND #4 AND #5 AND #6 AND #7

Supplementary Table S5. Characteristics of the studies included after screening for this meta-analysis.

Study	Hypertension, n (%)		Diabetes mellitus, n (%)		Hyper lipidaemia, n (%)		Obesity[#], n (%)		Peripheral artery disease, n (%)		COPD, n (%)		Previous MI, n (%)		Prior PCI		LVEF, n (%) or median (IQR) or mean±SD	
	SAG	MAG	SAG	MAG	SAG	MAG	SAG	MAG	SAG	MAG	SAG	MAG	SAG	MAG	SAG	MAG	SAG	MAG
Morris, 1990	NR	NR	NR	NR	NR	NR	NR	NR	NR	NR	NR	NR	6(1.43)	5(0.78)	NR	NR	0.51±0.10	0.49±0.10
Myers, 2000	38 (47)	46 (57)	12 (15)	11 (14)	44 (54)	55 (68)	NR	NR	NR	NR	NR	NR	12(14.8)	13(16)	NR	NR	62.60	60.80
Buxton, 2003	50(66)	39(59)	37(46)	27(37)	NR	NR	NR	NR	NR	NR	NR	NR	NR	NR	NR	NR	26 (32)[a]	22 (30)[a]
Muneretto, 2003	62(62)	60(60)	40(40)	41(41)	38(38)	40(40)	8(8)	6(6)	28(28)	31(31)	22(22)	19(19)	37(37)	41(41)	NR	NR	28(28)	29(29)
Muneretto, 2004	NR	NR	44(55)	41(51)	NR	NR	NR	NR	NR	NR	NR	NR	NR	NR	NR	NR	NR	NR
Collins, 2008	32(53)	46(56)	10(17)	15(18)	52 (87)	63 (77)	NR	NR	NR	NR	NR	NR	29(48)	45(55)	NR	NR	NR	NR
Nasso, 2008	NR	NR	78 (38)	227 (37)	NR	NR	28 (14)	84 (14)	NR	NR	57 (28)	165 (27)	61 (30)	178 (29)	NR	NR	28 (14)*	85 (14)*
Damgaard, 2009	81 (48)	82 (51)	43 (25)	39 (24)	147(86.5)	140(87)	28 ± 4	27 ± 4	NR	NR	NR	NR	NR	NR	NR	NR	42 (25)[b]	48 (30)[b]

**Goldman, 2011**	287 (78)	289 (79)	153 (42)	154 (42)	NA	NA	NR	NR	NR	NR	NR	NR	143 (39)	146 (40)	NR	NR	NR	NR
**Song, 2012**	21(84)	23(65.7)	13(52)	15(42.9)	11 (44)	17 (48.6)	7(28)	14(40)	NR	NR	NR	NR	NR	NR	11(44)	8(22.9)	3(13) [d]	0(0) [d]
**Le, 2015**	23(77)	22(73)	6(20)	9(30)	27(90)	25(83)	26(87)	27(90)	2(7)	3(10)	3(10)	2(7)	4(13)	2(7)	3(10)	3(10)	0(0) [c]	1(3) [c]
**Petrovic, 2015**	89 (89)	92 (92)	43 (43)	39 (39)	74 (74)	75 (75)	NR	NR	14 (14)	12 (12)	8 (8)	9 (9)	56 (56)	57 (57)	NR	NR	48 ± 11	49 ± 11
**Kim, 2018**	80 (71)	76 (68)	46 (41)	51 (46)	52 (46.4)	39 (34.8)	52 (46)	52 (46)	NR	NR	NR	NR	NR	NR	NR	NR	58 (54, 65)	57 (50, 63)
**Thujis, 2018**	523(76)	146(67)	198(30)	33(15)	477(70)	147(68)	237(34)	61(28)	69(10)	13(6)	62(9)	13(6)	103(15)	26(12)	NR	NR	57.0±8.8	59.0±9.6
**Buxton, 2020**	78 (70)	68 (60)	52 (46)	50 (44)	NR	NR	NR	NR	NR	NR	NR	NR	36 (32)	43 (38)	12 (11)	11 (10)	＞35%	＞35%
**Fomenko, 2021**	349(90.6)	362(93.5)	121(31.4)	129(33.3)	NR	NR	30.2±5.7	30.5±5.1	NR	NR	NR	NR	NR	NR	NR	NR	58.3% ±5.6%	58.5% ±5.1%
**Taggart, 2022 (dataset 1)**	852(78.6)	758(75.0)	253(23.3)	237(23.5)	1016(93.7)	949(94.0)	NR	NR	73(6.7)	72(7.1)	31(2.9)	25(2.5)	481(44.4)	398(39.4)	NR	NR	273(25.2)[a]	221(21.9)[a]
**Taggart, 2022 (dataset 2)**	852(78.6)	303(77.7)	253(23.3)	106(27.2)	1016(93.7)	364(93.3)	NR	NR	73(6.7)	21(5.4)	31(2.9)	6(1.5)	481(44.4)	161(41.3)	NR	NR	273(25.2)[a]	109(27.9)[a]
**Thujis, 2022**	742/990(74.9)	344/459(74.9)	361(36.1)	141(30.5)	751/985(76.2)	361/459(78.6)	310(31.0)	144(31.0)	128(12.8)	49(10.5)	89(8.9)	34(7.3)	351/984(35.7)	128/457(28.0)	NR	NR	NR	NR

NOTE: COPD, chronic obstructive pulmonary disease; IQR, interquartile range; LVEF, left ventricle ejection fraction; MAG, multiple arterial graft; MI, myocardial infarction; NR, not reported; PCI, percutaneous coronary intervention; SAG, single arterial graft; SD, standard deviation. [#] Body mass index (BMI)$\geqslant$ 25kg/m²; [a] [EF < 50%]; [b] [35% < EF < 49%]; [c] [EF<40%]; [d] [EF < 35%].

Supplementary Table S6. Baseline characteristics of patients in pooled studies.

Pooled studies	SAG	MAG	p-value
Male, n (%)	3694/4676 (79%)	3531/4404(80.18%)	0.164
Hypertension, n (%)	3307/4394(75.26%)	2756/3723(74.03%)	0.202
Diabetes mellitus, n (%)	1510/4676(32.29%)	1365/4404(30.99%)	0.184
Hyper lipidaemia, n (%)	2689/3451(77.92%)	2275/2783(81.75%)	0.0002
Obesity, n (%)	666/2158(30.86%)	388/1560(24.87%)	<0.0001
Peripheral artery disease, n (%)	314/3003(10.46%)	201/2312(8.69%)	0.031
COPD, n (%)	272/3205(8.49%)	273/2913(9.37%)	0.225
Previous MI, n (%)	1319/4244(31.08%)	1243/4199(29.60%)	0.14
Prior PCI, n (%)	26/167(15.57%)	22/156(12.36%)	0.389

NOTE: COPD, chronic obstructive pulmonary disease; MI, myocardial infarction; PCI, percutaneous coronary intervention.

Supplementary Table S7. Meta analysis for (A) all-cause mortality, (B) cardiac mortality, (C) myocardial infarction, (D) repeat revascularization, (E) stroke, (F) sternal wound complications and (G) bleeding complications with as-treated data.

**All-cause mortality**					
Subgroup	HR	95% Confidence interval		P value	I-square
		Lower Limit	Upper Limit		
RITA MAG vs.SAG	1.16	0.83	1.63	0.393	36.8
RA MAG vs. SAG	0.95	0.65	1.39	0.785	0
RITA/RA MAG vs.SAG	0.77	0.67	0.89	0.001	0
All	0.84	0.74	0.95	0.005	0
**Cardiac Mortality**					
Subgroup	HR	95% Confidence interval		P value	I-square
		Lower Limit	Upper Limit		
RITA MAG vs.SAG	0.6	0.24	1.49	0.273	0
RA MAG vs. SAG	1.57	0.44	5.6	0.488	0
RITA/RA MAG vs.SAG	0.81	0.64	1.01	0.059	0
All	0.81	0.65	1	0.051	0
**Myocardial Infarction**					
Subgroup	HR	95% Confidence interval		P value	I-square
		Lower Limit	Upper Limit		
RITA MAG vs.SAG	0.92	0.55	1.54	0.76	0
RA MAG vs. SAG	0.68	0.35	1.31	0.249	0
RITA/RA MAG vs.SAG	0.74	0.54	1.02	0.069	0
All	0.77	0.6	0.99	0.044	0
**Revascularization**					
Subgroup	HR	95% Confidence interval		P value	I-square
		Lower Limit	Upper Limit		

Subgroup	RR	95% Confidence interval: Lower Limit	Upper Limit	P value	I-square
RITA MAG vs.SAG	1.17	0.71	1.9	0.54	0
RA MAG vs. SAG	0.74	0.47	1.17	0.2	33.8
RITA/RA MAG vs.SAG	0.69	0.55	0.88	0.002	55.9
All	0.76	0.63	0.92	0.005	40.4

Stroke

Subgroup	RR	95% Confidence interval		P value	I-square
		Lower Limit	Upper Limit		
RITA MAG vs.SAG	0.66	0.32	1.39	0.274	0
RA MAG vs. SAG	0.99	0.42	2.31	0.981	0
RITA/RA MAG vs.SAG	0.86	0.61	1.2	0.369	0
All	0.84	0.63	1.12	0.229	0

Sternal wound complications

Subgroup	RR	95% Confidence interval		P value	I-square
		Lower Limit	Upper Limit		
RITA MAG vs.SAG	0.92	0.49	1.73	0.794	47.4
RA MAG vs. SAG	0.5	0.22	1.13	0.095	16.3
RITA/RA MAG vs.SAG	1.96	0.98	3.93	0.056	0
All	1.03	0.68	1.54	0.904	19.9

Bleeding

Subgroup	RR	95% Confidence interval		P value	I-square
		Lower Limit	Upper Limit		
RITA MAG vs.SAG	0.81	0.53	1.24	0.326	0
RA MAG vs. SAG	0.33	0.12	0.93	0.036	33.5
RITA/RA MAG vs.SAG	0.5	0.05	5.29	0.565	0
All	0.7	0.48	1.04	0.074	0

Supplementary Table S8. Subgroup analysis for (A) all-cause mortality, (B) cardiac mortality, (C) myocardial infarction, (D) repeat revascularization using intention to treat data.

All-cause mortality

Subgroup	HR	95% Confidence interval		P value	I-square (%)
		Lower Limit	Upper Limit		
＜5 years	0.96	0.7	1.33	0.818	9.9
5-10 years	1.17	0.73	1.89	0.509	0
＞10 years	0.78	0.68	0.9	0.001	0
All	0.83	0.73	0.94	0.004	0

Cardiac Mortality

Subgroup	HR	95% Confidence interval		P value	I-square (%)
		Lower Limit	Upper Limit		
＜5 years	0.63	0.35	1.14	0.127	0
5-10 years	1.33	0.29	6.06	0.711	0
＞10 years	0.83	0.66	1.05	0.112	0
All	0.81	0.65	1	0.05	0

Myocardial Infarction

Subgroup	HR	95% Confidence interval		P value	I-square (%)
		Lower Limit	Upper Limit		
＜5 years	0.67	0.44	1.02	0.061	0
5-10 years	0.71	0.22	2.29	0.571	0
＞10 years	0.84	0.6	1.16	0.29	0
All	0.77	0.59	0.99	0.039	0

Revascularization

Subgroup	HR	95% Confidence interval		P value	I-square (%)
		Lower Limit	Upper Limit		
＜5 years	0.71	0.49	1.02	0.063	52.6
5-10 years	0.87	0.47	1.59	0.643	50.9
＞10 years	0.76	0.6	0.97	0.029	0
All	0.76	0.63	0.92	0.004	38.9

Supplementary Table S9. Subgroup analysis for (A) all-cause mortality, (B) cardiac mortality, (C) myocardial infarction, (D) repeat revascularization using as-treat data.

Subgroup	HR	95% Confidence interval		P value	I-square (%)
		Lower Limit	Upper Limit		
**All-cause mortality**					
＜5 years	0.99	0.72	1.36	0.947	16.9
5-10 years	1.17	0.73	1.88	0.514	0
＞10 years	0.78	0.68	0.9	0.001	0
All	0.84	0.74	0.95	0.005	0
**Cardiac Mortality**					
Subgroup	HR	95% Confidence interval		P value	I-square (%)
		Lower Limit	Upper Limit		
＜5 years	0.63	0.35	1.15	0.135	0
5-10 years	1.33	0.29	6.03	0.714	0
＞10 years	0.83	0.66	1.05	0.112	0
All	0.81	0.65	1	0.051	0
**Myocardial Infarction**					
Subgroup	HR	95% Confidence interval		P value	I-square (%)
		Lower Limit	Upper Limit		
＜5 years	0.69	0.46	1.04	0.074	0
5-10 years	0.71	0.22	2.29	0.571	0
＞10 years	0.84	0.6	1.17	0.292	0
All	0.77	0.6	0.99	0.044	0
**Revascularization**					
Subgroup	HR	95% Confidence interval		P value	I-square (%)
		Lower Limit	Upper Limit		
＜5 years	0.72	0.5	1.04	0.08	54.8
5-10 years	0.86	0.47	1.59	0.638	50.6
＞10 years	0.76	0.6	0.97	0.028	0
All	0.76	0.63	0.92	0.005	40.4

Supplementary Table S10: Sensitivity analysis for intention to treat data.

**All-cause mortality**					
Deleted study	HR	95% Confidence interval		P value	I-square (%)
		Lower Limit	Upper Limit		
Morris, 1990	0.79	0.70	0.91	0.001	0.0
Myers, 2000	0.83	0.73	0.94	0.003	0.0
Buxton, 2003	0.82	0.73	0.94	0.003	0.0
Muneretto, 2003	0.83	0.73	0.95	0.005	0.0
Muneretto, 2004	0.83	0.73	0.94	0.004	0.0
Collins, 2008	0.83	0.73	0.94	0.004	0.0
Nasso, 2008	0.84	0.74	0.95	0.006	0.0
Damgaard, 2009	0.83	0.73	0.94	0.004	0.0
Goldman, 2011	0.83	0.73	0.94	0.003	0.0
Song, 2012	0.83	0.73	0.94	0.004	0.0
Le, 2015	0.83	0.73	0.94	0.004	0.0
Petrovic, 2015	0.83	0.73	0.94	0.004	0.0
Kim, 2018	0.82	0.73	0.94	0.003	0.0
Thujis, 2018	0.84	0.74	0.96	0.008	0.0
Buxton, 2020	0.83	0.73	0.95	0.006	0.0
Fomenko, 2021	0.83	0.73	0.94	0.004	0.0
Taggart, 2022 (dataset 1)	0.83	0.70	0.97	0.020	0.0
Taggart, 2022 (dataset 2)	0.86	0.75	0.98	0.025	0.0
Thujis, 2022	0.85	0.74	0.98	0.028	0.0
All	0.83	0.73	0.94	0.004	0.0
**Cardiac Mortality**					
Deleted study	HR	95% Confidence interval		P value	I-square (%)
		Lower Limit	Upper Limit		
Nasso, 2008	0.83	0.66	1.03	0.094	0.0
Goldman, 2011	0.80	0.64	0.99	0.043	0.0
Petrovic, 2015	0.80	0.64	0.99	0.043	0.0
Kim, 2018	0.81	0.65	1.00	0.049	0.0
Thujis, 2018	0.82	0.66	1.02	0.081	0.0

Taggart, 2022 (dataset 1)	0.76	0.56	1.01	0.061	0.0
Taggart, 2022 (dataset 2)	0.82	0.63	1.08	0.165	0.0
All	0.81	0.65	1.00	0.050	0.0

Myocardial Infarction

Deleted study	HR	95% Confidence interval		P value	I-square (%)
		Lower Limit	Upper Limit		
Myers, 2000	0.76	0.59	0.99	0.038	0.0
Muneretto, 2003	0.77	0.60	0.99	0.044	0.0
Muneretto, 2004	0.79	0.61	1.02	0.065	0.0
Nasso, 2008	0.81	0.63	1.06	0.121	0.0
Damgaard, 2009	0.77	0.60	1.00	0.048	0.0
Goldman, 2011	0.76	0.59	0.99	0.042	0.0
Song, 2012	0.77	0.59	0.99	0.040	0.0
Petrovic, 2015	0.77	0.59	0.99	0.042	0.0
Thujis, 2018	0.72	0.55	0.96	0.026	0.0
Buxton, 2020	0.76	0.59	0.99	0.043	0.0
Fomenko, 2021	0.77	0.60	0.99	0.044	0.0
Taggart, 2022 (dataset 1)	0.73	0.53	0.99	0.044	0.0
Taggart, 2022 (dataset 2)	0.75	0.57	1.00	0.049	0.0
All	0.77	0.59	0.99	0.039	0.0

Revascularization

Deleted study	HR	95% Confidence interval		P value	I-square (%)
		Lower Limit	Upper Limit		
Morris, 1990	0.75	0.62	0.91	0.004	42.4
Myers, 2000	0.75	0.62	0.91	0.003	37.5
Buxton, 2003	0.76	0.63	0.92	0.005	42.6
Muneretto, 2003	0.77	0.63	0.93	0.006	35.5
Muneretto, 2004	0.77	0.63	0.93	0.006	35.5
Collins, 2008	0.77	0.63	0.93	0.007	36.0
Nasso, 2008	0.81	0.66	0.98	0.033	18.3
Damgaard, 2009	0.75	0.62	0.91	0.003	41.3
Goldman, 2011	0.73	0.60	0.89	0.002	37.3
Song, 2012	0.76	0.63	0.92	0.006	42.1

Le, 2015	0.76	0.62	0.92	0.004	42.6
Petrovic, 2015	0.77	0.63	0.93	0.008	42.3
Kim, 2018	0.74	0.61	0.89	0.002	34.8
Thujis, 2018	0.74	0.61	0.91	0.004	41.8
Buxton, 2020	0.75	0.62	0.92	0.005	42.6
Taggart, 2022 (dataset 1)	0.72	0.56	0.92	0.008	41.8
Taggart, 2022 (dataset 2)	0.79	0.64	0.97	0.026	41.3
All	0.76	0.63	0.92	0.004	38.9

Stroke

Deleted study	RR	95% Confidence interval		P value	I-square (%)
		Lower Limit	Upper Limit		
Myers, 2000	0.83	0.62	1.11	0.211	0.0
Muneretto, 2003	0.84	0.63	1.12	0.233	0.0
Muneretto, 2004	0.84	0.63	1.13	0.251	0.0
Collins, 2008	0.84	0.63	1.13	0.242	0.0
Nasso, 2008	0.89	0.66	1.22	0.481	0.0
Damgaard, 2009	0.82	0.61	1.10	0.176	0.0
Goldman, 2011	0.81	0.60	1.10	0.172	0.0
Song, 2012	0.83	0.62	1.11	0.216	0.0
Le, 2015	0.82	0.61	1.10	0.178	0.0
Petrovic, 2015	0.82	0.61	1.10	0.183	0.0
Kim, 2018	0.84	0.62	1.12	0.235	0.0
Thujis, 2018	0.85	0.62	1.15	0.284	0.0
Fomenko, 2021	0.84	0.63	1.13	0.261	0.0
Taggart, 2022 (dataset 1)	0.83	0.57	1.22	0.347	0.0
Taggart, 2022 (dataset 2)	0.78	0.57	1.06	0.118	0.0
All	0.83	0.62	1.11	0.214	0.0

Sternal wound complications

Deleted study	RR	95% Confidence interval		P value	I-square (%)
		Lower Limit	Upper Limit		
Morris, 1990	0.81	0.76	1.84	0.453	10.5
Myers, 2000	0.97	0.65	1.47	0.897	11.8
Muneretto, 2003	1.02	0.68	1.54	0.918	24.5

Muneretto, 2004	1.02	0.68	1.54	0.918	24.5
Collins, 2008	1.18	0.77	1.82	0.443	3.1
Nasso, 2008	1.00	0.66	1.52	0.990	23.6
Damgaard, 2009	0.96	0.63	1.46	0.851	18.5
Goldman, 2011	0.97	0.64	1.47	0.896	17.6
Song, 2012	1.08	0.72	1.63	0.716	10.0
Le, 2015	1.02	0.68	1.54	0.918	24.5
Petrovic, 2015	1.04	0.69	1.58	0.838	23.0
Thujis, 2018	1.05	0.68	1.63	0.824	24.0
Buxton, 2020	1.04	0.69	1.57	0.845	23.1
Fomenko, 2021	0.96	0.62	1.47	0.842	21.1
Taggart, 2022 (dataset 1)	0.93	0.60	1.43	0.737	17.1
Taggart, 2022 (dataset 2)	0.95	0.62	1.46	0.815	19.9
All	1.02	0.68	1.53	0.919	19.1

Bleeding

Deleted study	RR	95% Confidence interval		P value	I-square (%)
		Lower Limit	Upper Limit		
Myers, 2000	0.71	0.48	1.06	0.097	16.0
Muneretto, 2003	0.71	0.48	1.05	0.085	17.0
Muneretto, 2004	0.68	0.46	1.01	0.056	6.7
Goldman, 2011	0.78	0.52	1.17	0.229	0.0
Song, 2012	0.73	0.49	1.08	0.117	0.0
Thujis, 2018	0.57	0.32	0.99	0.047	1.1
Fomenko, 2021	0.67	0.43	1.05	0.080	15.9
All	0.70	0.47	1.03	0.072	1.7

Supplementary Table S11: Sensitivity analysis for as-treated data.

All-cause mortality					
Deleted study	HR	95% Confidence interval		P value	I-square (%)
		Lower Limit	Upper Limit		
Morris, 1990	0.80	0.70	0.91	0.001	0.0
Myers, 2000	0.83	0.73	0.94	0.004	0.0
Buxton, 2003	0.83	0.73	0.94	0.004	0.0
Muneretto, 2003	0.84	0.74	0.95	0.006	0.0
Muneretto, 2004	0.84	0.74	0.95	0.006	0.0
Collins, 2008	0.84	0.74	0.95	0.006	0.0
Nasso, 2008	0.84	0.74	0.96	0.008	0.0
Damgaard, 2009	0.84	0.74	0.95	0.006	0.0
Goldman, 2011	0.83	0.73	0.94	0.004	0.0
Song, 2012	0.84	0.74	0.95	0.006	0.0
Le, 2015	0.84	0.74	0.95	0.005	0.0
Petrovic, 2015	0.83	0.73	0.95	0.005	0.0
Kim, 2018	0.83	0.73	0.94	0.004	0.0
Thujis, 2018	0.85	0.75	0.96	0.011	0.0
Buxton, 2020	0.84	0.74	0.95	0.007	0.0
Fomenko, 2021	0.84	0.74	0.95	0.006	0.0
Taggart, 2022 (dataset 1)	0.83	0.71	0.98	0.028	0.0
Taggart, 2022 (dataset 2)	0.86	0.75	0.99	0.033	0.0
Thujis, 2022	0.86	0.75	0.99	0.036	0.0
All	0.84	0.74	0.95	0.005	0.0
**Cardiac Mortality**					
Deleted study	HR	95% Confidence interval		P value	I-square (%)
		Lower Limit	Upper Limit		
Nasso, 2008	0.83	0.66	1.03	0.096	0.0
Goldman, 2011	0.80	0.64	0.99	0.043	0.0
Petrovic, 2015	0.80	0.64	0.99	0.044	0.0
Kim, 2018	0.81	0.65	1.00	0.051	0.0

Thujis, 2018	0.82	0.66	1.03	0.083	0.0
Taggart, 2022 (dataset 1)	0.76	0.56	1.02	0.063	0.0
Taggart, 2022 (dataset 2)	0.83	0.63	1.09	0.169	0.0
All	0.81	0.65	1.00	0.051	0.0

Myocardial Infarction

Deleted study	HR	95% Confidence interval		P value	I-square (%)
		Lower Limit	Upper Limit		
Myers, 2000	0.77	0.60	0.99	0.043	0.0
Muneretto, 2003	0.78	0.60	1.00	0.049	0.0
Muneretto, 2004	0.79	0.61	1.02	0.072	0.0
Nasso, 2008	0.82	0.63	1.06	0.132	0.0
Damgaard, 2009	0.78	0.60	1.00	0.053	0.0
Goldman, 2011	0.77	0.59	0.99	0.044	0.0
Song, 2012	0.77	0.60	0.99	0.044	0.0
Petrovic, 2015	0.77	0.60	1.00	0.047	0.0
Thujis, 2018	0.73	0.55	0.97	0.029	0.0
Buxton, 2020	0.77	0.59	1.00	0.047	0.0
Fomenko, 2021	0.78	0.60	1.00	0.049	0.0
Taggart, 2022 (dataset 1)	0.74	0.54	1.00	0.051	0.0
Taggart, 2022 (dataset 2)	0.76	0.58	1.01	0.055	0.0
All	0.77	0.60	0.99	0.044	0.0

Revascularization

Deleted study	HR	95% Confidence interval		P value	I-square (%)
		Lower Limit	Upper Limit		
Morris, 1990	0.75	0.62	0.92	0.004	43.7
Myers, 2000	0.75	0.62	0.91	0.003	39.2
Buxton, 2003	0.76	0.63	0.92	0.005	44.0
Muneretto, 2003	0.77	0.63	0.93	0.007	37.2
Muneretto, 2004	0.77	0.63	0.93	0.007	37.2
Collins, 2008	0.77	0.64	0.93	0.008	37.6
Nasso, 2008	0.81	0.67	0.99	0.037	20.9
Damgaard, 2009	0.75	0.62	0.91	0.004	42.6

Goldman, 2011	0.73	0.60	0.89	0.002	37.3
Song, 2012	0.77	0.63	0.93	0.007	43.4
Le, 2015	0.76	0.63	0.92	0.005	44.0
Petrovic, 2015	0.77	0.63	0.94	0.009	43.7
Kim, 2018	0.74	0.61	0.90	0.002	36.7
Thujis, 2018	0.74	0.61	0.91	0.004	43.2
Buxton, 2020	0.76	0.62	0.92	0.005	44.1
Taggart, 2022 (dataset 1)	0.72	0.56	0.92	0.010	43.3
Taggart, 2022 (dataset 2)	0.79	0.64	0.98	0.029	42.6
All	0.76	0.63	0.92	0.005	40.4

Stroke

Deleted study	RR	95% Confidence interval		P value	I-square (%)
		Lower Limit	Upper Limit		
Myers, 2000	0.84	0.62	0.12	0.226	0.0
Muneretto, 2003	0.84	0.63	1.13	0.249	0.0
Muneretto, 2004	0.85	0.63	1.14	0.268	0.0
Collins, 2008	0.85	0.63	0.13	0.259	0.0
Nasso, 2008	0.90	0.66	1.23	0.508	0.0
Damgaard, 2009	0.82	0.61	1.10	0.187	0.0
Goldman, 2011	0.81	0.60	1.10	0.173	0.0
Song, 2012	0.84	0.63	1.12	0.232	0.0
Le, 2015	0.82	0.62	1.10	0.193	0.0
Petrovic, 2015	0.82	0.61	1.11	0.197	0.0
Kim, 2018	0.84	0.63	1.13	0.252	0.0
Thujis, 2018	0.85	0.63	1.16	0.303	0.0
Fomenko, 2021	0.85	0.63	1.14	0.278	0.0
Taggart, 2022 (dataset 1)	0.84	0.58	1.23	0.375	0.0
Taggart, 2022 (dataset 2)	0.79	0.58	1.07	0.128	0.0
All	0.84	0.63	1.12	0.229	0.0

Sternal wound complications

Deleted study	RR	95% Confidence interval		P value	I-square (%)
		Lower Limit	Upper Limit		

Morris, 1990	1.19	0.77	1.84	0.446	12.0
Myers, 2000	0.98	0.65	1.47	0.912	12.8
Muneretto, 2003	1.03	0.68	1.55	0.903	25.2
Muneretto, 2004	1.03	0.68	1.54	0.903	25.2
Collins, 2008	1.19	0.77	1.83	0.432	4.1
Nasso, 2008	1.01	0.67	1.52	0.975	24.4
Damgaard, 2009	0.96	0.63	1.46	0.861	19.1
Goldman, 2011	0.97	0.65	1.47	0.901	17.6
Song, 2012	1.08	0.72	1.63	0.703	11.0
Le, 2015	1.03	0.68	1.54	0.900	25.2
Petrovic, 2015	1.05	0.70	1.58	0.824	23.7
Thujis, 2018	1.06	0.68	1.63	0.808	24.7
Buxton, 2020	1.05	0.69	1.58	0.827	23.8
Fomenko, 2021	0.96	0.63	1.48	0.858	21.9
Taggart, 2022 (dataset 1)	0.93	0.61	1.44	0.753	18.1
Taggart, 2022 (dataset 2)	0.95	0.62	1.46	0.831	20.7
All	1.03	0.68	1.54	0.904	19.9

Bleeding

Deleted study	RR	95% Confidence interval		P value	I-square (%)
		Lower Limit	Upper Limit		
Myers, 2000	0.71	0.48	1.07	0.099	12.1
Muneretto, 2003	0.71	0.48	1.05	0.087	13.3
Muneretto, 2004	0.68	0.46	1.01	0.057	2.1
Goldman, 2011	0.78	0.52	1.17	0.229	0.0
Song, 2012	0.73	0.49	1.09	0.120	0.0
Thujis, 2018	0.57	0.33	1.00	0.050	0.0
Fomenko, 2021	0.67	0.43	1.05	0.083	12.2
All	0.70	0.48	1.04	0.074	0.0

Supplementary Table S12. Subgroup analysis for (A) all-cause mortality, (B) cardiac mortality, (C) myocardial infarction, (D) repeat revascularization, (E) stroke, (F) sternal wound complications and (G) bleeding complications with as-treated data.

**All-cause mortality**					
Subgroup	HR	95% Confidence interval		P value	I-square
		Lower Limit	Upper Limit		
RCT	0.87	0.75	1.00	0.053	0.00
RCT post-hoc	0.71	0.54	0.93	0.012	0.00
All	0.83	0.73	0.94	0.004	0.00
**Cardiac Mortality**					
Subgroup	HR	95% Confidence interval		P value	I-square
		Lower Limit	Upper Limit		
RCT	0.82	0.66	1.03	0.081	0.00
RCT post-hoc	0.57	0.22	1.48	0.247	0.00
All	0.81	0.65	1.00	0.050	0.00
**Myocardial Infarction**					
Subgroup	HR	95% Confidence interval		P value	I-square
		Lower Limit	Upper Limit		
RCT	0.73	0.55	0.97	0.029	0.00
RCT post-hoc	0.95	0.54	1.66	0.847	0.00
All	0.77	0.60	0.99	0.042	0.00
**Revascularization**					
Subgroup	RR	95% Confidence interval		P value	I-square
		Lower Limit	Upper Limit		
RCT	0.74	0.61	0.91	0.004	41.80
RCT post-hoc	0.92	0.50	1.71	0.801	0.00
All	0.76	0.63	0.92	0.004	38.90
**Stroke**					
Subgroup	RR	95% Confidence interval		P value	I-square
		Lower Limit	Upper Limit		
RCT	0.85	0.63	1.15	0.284	0.00

Subgroup	RR	95% Confidence interval		P value	I-square
		Lower Limit	Upper Limit		
RCT post-hoc	0.69	0.26	1.84	0.457	0.00
All	0.83	0.62	1.11	0.214	0.00

Sternal wound complications

Subgroup	RR	95% Confidence interval		P value	I-square
		Lower Limit	Upper Limit		
RCT	1.05	0.68	1.63	0.823	24.00
RCT post-hoc	0.85	0.28	2.58	0.774	0.00
All	1.02	0.68	1.53	0.918	19.10

Bleeding

Subgroup	RR	95% Confidence interval		P value	I-square
		Lower Limit	Upper Limit		
RCT	0.56	0.32	0.99	0.046	0.50
RCT post-hoc	0.85	0.50	1.47	0.563	0.00
All	0.70	0.47	1.03	0.072	1.70
